# Activation of Human Monocytes by Live *Borrelia burgdorferi* Generates TLR2-Dependent and -Independent Responses Which Include Induction of IFN-β

**DOI:** 10.1371/journal.ppat.1000444

**Published:** 2009-05-22

**Authors:** Juan C. Salazar, Star Duhnam-Ems, Carson La Vake, Adriana R. Cruz, Meagan W. Moore, Melissa J. Caimano, Leonor Velez-Climent, Jonathan Shupe, Winfried Krueger, Justin D. Radolf

**Affiliations:** 1 Connecticut Children's Medical Center, Division of Pediatric Infectious Diseases, Hartford, Connecticut, United States of America; 2 Department of Pediatrics, University of Connecticut Health Center, Farmington, Connecticut, United States of America; 3 Department of Medicine, University of Connecticut Health Center, Farmington, Connecticut, United States of America; 4 Centro Internacional de Entrenamiento e Investigaciones Medicas, Cali, Colombia; 5 Department of Genetics and Developmental Biology, University of Connecticut Health Center, Farmington, Connecticut, United States of America; Stanford University, United States of America

## Abstract

It is widely believed that innate immune responses to *Borrelia burgdorferi* (Bb) are primarily triggered by the spirochete's outer membrane lipoproteins signaling through cell surface TLR1/2. We recently challenged this notion by demonstrating that phagocytosis of live Bb by peripheral blood mononuclear cells (PBMCs) elicited greater production of proinflammatory cytokines than did equivalent bacterial lysates. Using whole genome microarrays, we show herein that, compared to lysates, live spirochetes elicited a more intense and much broader transcriptional response involving genes associated with diverse cellular processes; among these were IFN-β and a number of interferon-stimulated genes (ISGs), which are not known to result from TLR2 signaling. Using isolated monocytes, we demonstrated that cell activation signals elicited by live Bb result from cell surface interactions and uptake and degradation of organisms within phagosomes. As with PBCMs, live Bb induced markedly greater transcription and secretion of TNF-α, IL-6, IL-10 and IL-1β in monocytes than did lysates. Secreted IL-18, which, like IL-1β, also requires cleavage by activated caspase-1, was generated only in response to live Bb. Pro-inflammatory cytokine production by TLR2-deficient murine macrophages was only moderately diminished in response to live Bb but was drastically impaired against lysates; TLR2 deficiency had no significant effect on uptake and degradation of spirochetes. As with PBMCs, live Bb was a much more potent inducer of IFN-β and ISGs in isolated monocytes than were lysates or a synthetic TLR2 agonist. Collectively, our results indicate that the enhanced innate immune responses of monocytes following phagocytosis of live Bb have both TLR2-dependent and -independent components and that the latter induce transcription of type I IFNs and ISGs.

## Author Summary

Lyme disease is a tick-borne infectious disorder caused by the spirochetal pathogen *Borrelia burgdorferi* (Bb). Innate immune responses to Bb are thought to be triggered by the spirochete's outer membrane lipoproteins signaling through cell surface toll-like receptors (TLR1/2). Using a whole genome microarray technique, we showed that live spirochetes elicited a more intense and broader immune response in human peripheral blood mononuclear cells (PBMCs) than could be explained simply by TLR1/2 cell surface stimulation. Of particular interest, live Bb also uniquely induced transcription of type I interferons. In similarly stimulated isolated human monocytes, live Bb generated a greater production of pro- and anti-inflammatory cytokines (TNF-α, IL-6, IL-10 and IL-1β), as well as interferon-β (IFN-β). Secreted IL-18, which like IL-1β requires cytosolic cleavage of its inactive form by activated caspase-1, was generated only in response to live Bb. The cytosolic responses occurred despite evidence that phagocytosed spirochetes were rapidly degraded in phagosomal vacuoles, and unable to escape unscathed into the cell cytosol. We conclude that the innate immune signals generated in human monocytes by phagocytosed spirochetes allow the host to control the bacterium through a number of non-exclusive pathways, that are both TLR2-dependent and -independent, and include a type I interferon response.

## Introduction

Lyme disease (LD), the most commonly reported vector-borne illness in the United States, is a tick-borne, multi-system, inflammatory, infectious disorder caused by the spirochetal bacterium *Borrelia burgdorferi* (Bb) [1]. The disease is often heralded in its early stage by erythema migrans (EM), an expanding annular rash which develops following inoculation of spirochetes into the skin at the site of tick feeding and is frequently accompanied by ‘flu like’ symptoms, including myalgias, arthralgias, and fever [1]–[3]. If treated appropriately, the prognosis is excellent [3],[4]; however, if untreated, hematogenous dissemination of spirochetes may give rise to a wide range of clinical manifestations, most commonly involving the central nervous system, joints and heart [1]. Within days of treatment, the signs and symptoms associated with the disease typically begin to subside, although in some individuals a complete recovery can take several weeks or even months [5]. A minority of treated patients may go on to develop a poorly defined fibromyalgia-like illness, which is not responsive to prolonged antimicrobial therapy [6],[7]. Understanding the ontogeny of the immune response to the bacterium may provide insights into why some patients remain persistently symptomatic while others recover more rapidly.

Until recently, most efforts to understand how Bb initiates innate immune cell activation have focused on the pro-inflammatory attributes of spirochetal lipoproteins [8]–[15], while less has been done to define the mechanisms underlying immune recognition elicited by live spirochetes. The emphasis on borrelial lipoproteins (BLPs) as innate immune agonists emerged from the discovery that spirochetes expresses an abundance of these molecules, many on their outer membrane, and that the borrelial cell envelope lacks the potent gram negative proinflammatory glycolipid, lipopolysaccharide (LPS) [16]–[19]. Unlike LPS, which signals through the pattern recognition receptors (PRRs) Toll-like receptor (TLR)-4 and CD14 [20], lipoproteins signal through TLR1/2 heterodimers [10], [13], [21]–[23], also in a CD14 dependent manner [12],[24],[25]. Intradermal injection of spirochetal lipoprotein analogs (lipopeptides), in both animals [8] and humans [15],[26], confirmed *in situ* that BLPs indeed have the capability to activate macrophages and induce dendritic cell (DC) maturation. Collectively, these prior studies led to the viewpoint that innate immune cell activation in LD occurs predominantly through the interaction of spirochetal lipoproteins with CD14 and/or TLR1/2 on the surfaces of macrophages and DCs.

The findings that experimentally infected mice deficient in CD14 [25],[27], TLR2 [14],[28] developed even more severe arthritis than their wild type counterparts, and that mice lacking the TLR adapter protein myeloid differentiation factor 88 (MYD88) [29] also developed arthritis, provided the first clear indication that intact spirochetes may employ additional TLRs and/or TLR-independent pathways to induce acute inflammation. Further evidence that spirochetes generate TLR2 independent signals in both humans and mice has been obtained using *ex vivo* stimulation models. Behera *et al* [30] demonstrated that blocking of TLR2 on the surface of human chondrocytes eliminated the inflammatory response to purified lipoproteins but not to intact spirochetes. The same group [31] recently confirmed using a mouse model that proinflammatory cytokine production was only partially reduced in TLR2 deficient bone marrow derived macrophages (BMDMs) stimulated with live Bb and showed that maximal TLR2 stimulation is dependent on phagocytosis. Using human peripheral blood mononuclear cells (PBMCs) we previously provided evidence that phagocytosed live bacteria initiate activation programs in monocytes and DCs that differ both quantitatively and qualitatively from those evoked by cell surface-mediated signals generated by lipoprotein rich bacterial lysates [32],[33]. The marked increases in IL-1β secretion in response to live bacteria were indicative that phagocytosis of the spirochete generates phagolysosomal signals that result in much greater activation of caspase-1 than could be elicited by cell surface mediated TLR1/2 stimulation by bacterial lysates. In the same experiments heat killed spirochetes were readily phagocytosed and also induced a stronger pro-inflammatory response than bacterial lysates. The greater complexity of the signaling events triggered by internalized spirochetes was further underscored by their ability to induce programmed cell death responses in monocytes.

The current study was conducted to further elucidate the multifaceted mechanisms by which live Bb initiates and maintains innate immune responses in human monocytes. We focused on monocytes because of the seminal role these cells have in the recognition and killing of spirochetes during the course of LD [34]–[39]. We began by using microarray methods to compare the global transcriptional responses elicited in PBMCs by live Bb and equivalent amounts of borrelial lysates. The arrays provided unambiguous evidence that live bacteria elicit a proinflammatory response that is not just more intense but also broader, involving genes associated with diverse cellular processes including immune activation, ion transport, protein ubiquitination, and cell damage and repair. The finding that live bacteria induced transcription of interferon β (IFN-β), along with a number of Type I interferon-stimulated genes (ISGs), was of particular relevance given that TLR2-derived signals alone cannot induce type I interferons [40],[41]. Interestingly, type I interferons have recently been shown to have an important role in the development of arthritis in a murine LD model [42],[43]. Using isolated human monocytes, we demonstrated that this cell is indeed the primary source of the many unique inflammatory signals engendered by live Bb, and that these signaling pathways, including activation of caspase-1 and induction of IFN-β and associated genes, arise under conditions in which spirochetes manifest no capacity to escape vacuolar confinement and degradation. Collectively our results highlight the ability of LD spirochetes to induce diverse signals which coincide with degradation of spirochetes within phagolysosomes that are both stronger and distinct from those generated by spirochetal lipoproteins and which cannot be entirely attributed to the canonical prototypic TLR1/2 dependent signaling.

## Results

### Identification of distinct PBMC transcriptional responses to live vs. lysed *Borrelia burgdorferi*

We previously showed in a PBMC-Bb stimulation model that phagocytosed live bacteria initiate activation programs in monocytes and dendritic cells (DCs) that differ both quantitatively and qualitatively from those evoked by cell surface-mediated signals generated by equivalent amounts of lipoprotein rich bacterial lysates [32],[33]. We also demonstrated that abrogation of phagocytosis by Cytochalasin D diminished the production of inflammatory cytokines by live bacteria to levels comparable to those induced by lysates [32]. In this study, we used a whole genome microarray method to more completely characterize the transcriptional responses generated by live or lysed spirochetes in the PBMC-Bb stimulation model. Equivalence between input live and lysed Bb used for each individual experiment was confirmed by SDS-PAGE and silver staining (data not shown). PBMCs also were incubated with fluorescent microspheres (beads) to evaluate the transcriptional events associated with the cytoskeletal changes that are known to follow the binding and internalization of inert particles [44]. Gene intensity values generated in response to each of the three conditions studied then were compared to those from unstimulated cells.

**Figure 1:**
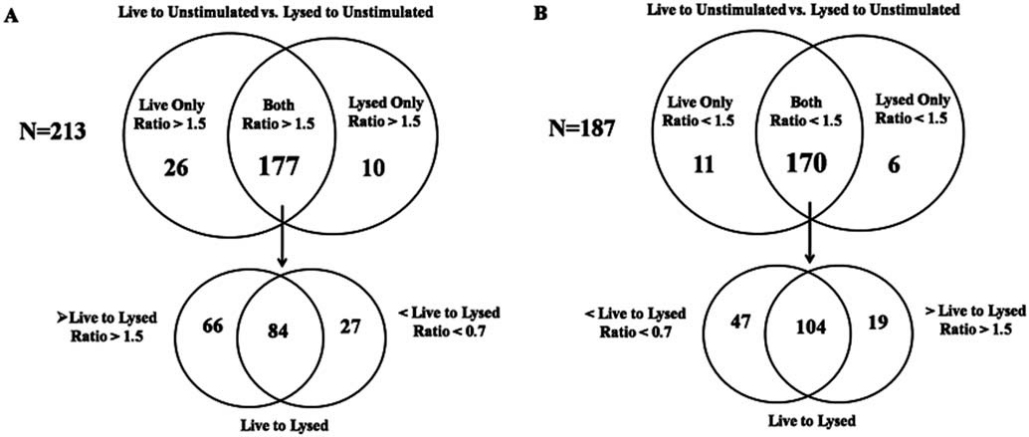
Distinct gene expression profiles obtained from human PBMCs stimulated with either live or lysed *Borrelia burgdorferi*. Genes whose normalized intensity values were deemed to be statistically significant in the PBMC array, and had a known biologic function, were used to determine the total number of genes whose intensity values were proportionately higher or lower in PBMCs in response to either live Bb and/or lysed Bb vs. unstimulated conditions. The top half of the Venn diagrams show the total number of gene transcripts that were more intensely or exclusively regulated by live and/or lysed Bb vs. unstimulated conditions (N = 213 genes) (A), or less intensely or exclusively down-regulated by live and/or lysed Bb vs. unstimulated cells (N = 187) (B). For similarly up-regulated (N = 177) or down-regulated (N = 170) genes, the arrows point down to a direct comparative analysis between live or lysed Bb generated gene intensity values. Genes in this category were further stratified depending on whether or not they were proportionately higher (live to lysed ratio >1.5), lower (live to lysed ratio <1.5) or equal between live vs lysed Bb stimulated cells.

The statistical analysis revealed that 529 genes were differentially regulated in one or more of the three conditions studied (live and lysed Bb and beads). Of the total group of 529 genes, 400 encode molecules with identified biologic functions, and these were selected for further analysis. Based on a ratio determined by directly comparing individual gene transcript intensity values generated from beads, live and lysed vs. unstimulated PBMCs, we considered genes to be either differentially up-regulated (ratio >1.5), differentially down-regulated (ratio <0.7) or unchanged. With few exceptions, the transcriptional profile of PBMCs stimulated with beads was very similar to that obtained from unstimulated cells. In contrast, 213 of the 400 genes studied were differentially up-regulated (**Fig. 1A**) and 187 were down-regulated (**Fig. 1B**) in response to either live and/or lysed Bb. Genes within this group of 400 were then classified into four different sub-groups. The first, designated the “core group”, was comprised of 184 genes whose intensity values compared to unstimulated cells were similarly up- or down-regulated in response to either live or lysed spirochetes (**Table S1**). The second group included genes classified as being either more intensely or exclusively up-regulated (**Table 1**) in response to live Bb. The third group contained genes more intensely down-regulated in response to live spirochetes, in some cases exclusively in response to live bacteria (**Table S2**). The final group consisted of a small number of genes that were either strongly or solely up-regulated in response to borrelial lysates (**Table S3**).

**Table 1:** Genes classified as being either more intensely or exclusively up-regulated in peripheral blood mononuclear cells (PBMCs) stimulated with live *Borrelia burgdorferi* (Bb) MOI (10∶1) in comparison to similar concentrations of borrelial lysates.

Gene Number	Annotation	Beads/UN	Live/UN	Lysate/UN	Live/Lysate	Description/Function
						**Cytokines/Chemokines**
NM_000619.2	IFNG	1.02	88.34	2.10	42.11	Interferon gamma
NM_001874.3	CPM	1.00	75.79	1.98	38.27	GMCSF
NM_002187.2	IL12B	0.78	34.90	2.50	13.96	IL-12p40 subunit of IL-12 and IL-23
NM_000594.2	TNF	0.76	35.29	3.98	8.87	Tumor necrosis factor-α
NM_172200.1	IL15RA	0.42	119.68	36.23	3.30	IL-15Rα receptor
NM_019618.2	IL1F9	1.14	56.89	18.73	3.04	Interleukin 1 family, member 9
NM_000758.2	CSF2	2.55	148.26	49.90	2.97	GCSF
NM_021006.4	CCL3L1	0.82	39.84	15.24	2.61	Chemokine (C-C motif) ligand 3-like 1
NM_002984.1	CCL4	1.93	31.98	12.48	2.56	Chemokine (C-C motif) ligand 4-MIP1B
NM_013371.2	IL19	1.91	48.50	19.70	2.46	Interleukin 19
NM_000600.1	IL6	0.09	1228.77	582.05	2.11	Interleukin 6
NM_173842.1	IL1RN	0.25	27.51	15.07	1.83	Interleukin 1 receptor antagonist
NM_001001437.2	CCL3L3	0.69	28.84	17.56	1.64	Chemokine (C-C motif) ligand 3-like 3
NM_002983.1	CCL3	0.74	31.96	19.73	1.62	Macrophage inflammatory protein-1
NM_000575.3	IL1A	0.67	32.67	21.38	1.53	Interleukin 1, α
						**Signaling/transcription/regulation**
NM_002728.4	**PRG2**	5.40	29.36	0.57	51.77	Integral membrane proteins
NM_005544.1	IRS1	0.82	49.63	2.11	23.54	Insulin receptor substrate
NM_014344.2	FJX1	1.00	190.81	9.07	21.04	Transmembrane protein may be involved in signaling
NM_002759.1	EIF2AK2	1.10	9.49	1.61	5.91	Eukaryotic translation initiation factor 2-alpha kinase 2
NM_139266.1	STAT1	0.98	12.33	3.05	4.04	Signal Transducer of Activation-1
NM_018323.2	PI4K2B	1.14	7.58	2.22	3.41	Phosphatidylinositol 4-kinase
NM_016457.3	**PRKD2**	1.13	2.44	0.77	3.16	Serine- and threonine-specific protein kinase
NM_004688.1	**NMI**	0.93	2.88	1.17	2.46	Augments cytokine-mediated STAT transcription.
NM_000963.1	COX2	1.02	52.42	21.71	2.41	Cyclooxygenase 2
NM_001005474.1	NFKBIZ	0.89	3.56	1.95	1.83	Inhibits NF-kB activity
NM_004419.3	DUSP5	0.95	8.63	4.87	1.77	Protein-tyrosine phosphatase family
NM_021181.3	SLAMF7	0.82	7.75	4.72	1.64	Mediates NK cell activation
NM_006724.2	MAPKKK4	0.98	3.67	2.44	1.50	Protein kinase signal transduction cascade
						**Type I Interferons**
NM_139174.2	LOC129607	1.00	246.25	5.82	42.31	Novel interferon-β-induced gene
NM_080657.3	RSAD2	0.58	648.97	24.69	26.28	Interferon-induced protein (Viperin)
NM_001549.2	IFIT3	1.25	54.49	3.87	14.08	Interferon-induced protein
NM_001032409.1	**OAS1**	1.11	12.70	0.99	12.79	Oligoadenylate synthetase 1
NM_001547.3	IFIT2	1.00	19.05	1.53	12.42	Interferon-induced protein 2
NM_001548.2	IFIT1	1.00	19.05	1.53	12.42	Interferon-induced protein 1
NM_005101.1	ISG15	1.02	23.46	1.89	12.42	Ubiquitin-like protein
NM_006187.2	OAS3	0.86	23.43	2.26	10.37	Oligoadenylate synthetase 3
NM_002176.2	IFNB1	0.74	47.44	4.73	10.03	Interferon-β
NM_002462.2	MX1	1.03	22.38	2.28	9.80	Interferon-induced GTPase
NM_006417.2	**IFI44**	1.02	12.31	1.42	8.65	Interferon alpha inducible protein
NM_199139.1	BIRC4BP	0.86	9.95	1.62	6.13	Interferon stimulated inhibitor of apoptosis
NM_002535.2	OAS2	1.21	11.56	1.99	5.80	Oligoadenylate synthetase 2
NM_022168.2	**IFIH1**	0.89	7.71	1.46	5.27	Interferon- induced with helicase C domain 1
NM_002053.1	GBP1	0.78	9.55	1.86	5.13	Interferon-induced GTPase
NM_005533.2	**IFI35**	0.99	7.12	1.45	4.91	Interferon-induced protein 35
NM_004030.1	**IRF7**	1.11	5.79	1.20	4.81	Interferon regulatory factor 7
NM_207315.1	**ISG20**	1.15	5.36	1.24	4.33	Interferon-stimulated exonuclease
NM_005531.1	IFI16	1.14	4.20	1.56	2.69	Interferon-induced protein 16
NM_006084.3	**ISGF3G**	1.00	2.91	1.49	1.95	Interferon-stimulated transcription factor 3 (IRF9)
						**Cell activation/cell cycle**
NM_017654.2	**SAMD9**	0.67	15.31	0.33	46.75	Sterile alpha motif domain 9
NM_033405.2	**PRIC285**	1.09	12.47	1.49	8.35	Transcriptional coactivator
NM_001781.1	CD69	1.02	8.37	2.25	3.71	Inducible cell surface glycoprotein
NM_152542.2	**PPM1K**	0.93	4.05	1.09	3.71	Protein serine/threonine phosphatase complex
NM_001993.2	F3	1.00	641.36	173.16	3.70	Thromboplastin (coagulation factor 3)
NM_014398.2	LAMP3	1.05	16.13	5.06	3.19	Lysosomal-associated membrane protein 3
NM_002662.2	PLD1	2.51	135.58	45.22	3.00	Phospholipase.
NM_033379.2	CDC2	1.00	73.47	31.17	2.36	Member of the Ser/Thr protein kinase family
NM_000777.2	CYP3A5	2.40	73.16	33.92	2.16	Cytochrome P450
NM_015714.2	G0S2	0.15	37.50	19.12	1.96	Commits cells to G1 phase of cell cycle
NM_005807.2	PRG4	0.72	78.96	47.05	1.68	Novel hematopoietic growth factor
						**Protein Ubiquitination. Ubiqutin like**
NM_017414.2	USP18	0.74	84.98	1.71	49.83	Protease that specifically removes ISG15
NM_016323.1	HERC5	1.19	23.77	2.46	9.66	IFN-induced HECT-type E3 protein ligase.
NM_001013005.1	HERC6	1.09	14.09	1.81	7.77	E3 ubiquitin-protein ligase
NM_033092.1	TRIM5	0.68	28.11	5.86	4.79	E3 ubiquitin ligase
NM_020760.1	HECW2	1.20	52.56	16.63	3.16	E3 ubiquitin-protein ligase
NM_004223.3	**UBE2L6**	1.00	3.61	1.30	2.76	Major ISG15-conjugating enzyme
						**Cell Damage and Repair**
NM_009587.1	**LGALS9**	1.08	3.25	0.05	62.34	Galectin9
NM_031458.1	PARP9	0.94	12.81	3.14	4.08	poly (ADP-ribose) polymerase family, member 9
NM_015907.2	**LAP3**	0.96	4.61	1.14	4.03	Leucine aminopeptidase. IFN γ-induced apotosis
NM_017554.1	**PARP14**	1.04	4.71	1.26	3.74	poly (ADP-ribose) polymerase family, member 14
NM_032789.1	**PARP10**	1.17	4.09	1.13	3.63	poly (ADP-ribose) polymerase family, member 10
NM_006528.2	TFPI2	1.00	51.73	14.28	3.62	Caspase-mediated, pro-apoptotic signaling.
NM_022750.2	**PARP12**	0.86	4.26	1.28	3.34	poly (ADP-ribose) polymerase family, member 12
NM_015675.1	GADD45B	0.98	4.84	2.00	2.42	Growth Arrest and DNA-damage-inducible β
NM_003580.2	**NSMAF**	0.99	2.90	1.48	1.96	Neutral sphingomyelinase activation associated factor
NM_001024688.1	NBN	0.92	2.66	1.69	1.58	Involved in DNA damage and repair
						**Metal Binding / Ion Transport Systems**
NM_022003.1	**FXYD6**	2.11	22.49	0.10	224.26	Regulator of of Na-K ATPase Channel
NM_024625.3	**ZC3HAV1**	0.77	2.93	1.09	2.70	Metal (Zn) and RNA binding
NM_021035.1	**ZNFX1**	0.87	2.98	1.29	2.31	Metal binding Zinc Finger Protein
NM_016119.1	**PHF11**	1.08	2.78	1.35	2.05	Metal binding Zinc Finger Protein
NM_005953.2	MT2A	0.89	6.62	3.38	1.96	Metallothionein
NM_153259.2	MCOLN2	0.94	4.95	2.97	1.67	Mucolipin
						**Protein Transport**
NM_002959.4	SORT1	0.59	72.56	19.9	4.67	Trans-Golgi network (TGN) transmembrane protein
NM_016410.2	**CHMP5**	0.93	2.28	1.13	2.02	Chromatin-modifying protein
NM_003666.2	**BLZF1**	0.99	2.02	1.14	1.78	Golgin

Values shown correspond to a ratio determined between normalized gene intensity values obtained after a four-hour PBMC stimulation with either inert beads, live or lysed Bb (MOI 10∶1) in proportion to gene intensity values from unstimulated cells. Annotated names in **bold** letters correspond to genes that were exclusively up-regulated by live Bb.

### Core transcriptional responses are largely representative of cell surface TLR signals

The “core group” of genes provided a snapshot of the various innate immune signaling pathways which are triggered from the cell surface in response to intact spirochetes or spirochetal constituents (lysates) (**Table S1**). Based on prior work, we considered several of these genes to be largely representative of downstream TLR2-mediated signals. Within this core group there also were several chemokine associated genes, including IL-8 and CCL2. IL-8 has been shown to be secreted in response to TLR1/2 mediated signals induced by either spirochetal lipoproteins [45],[46] or Bb lysates [47]. Moreover, synthesis of this chemokine in response to borrelial lysates can be partially abrogated by TLR2 blockade [47]. The TLR-induced neutrophil chemoattractant CCL2 (also known as MCP-1) is over expressed in LD erythema migrans (EM) dermal infiltrates [48] and in joint tissues from Lyme arthritis susceptible mice [49]. The gene for the suppressor of cytokine signaling protein 3 (SOCS3), a negative regulator of cytokines that signal through the Janus kinase/signal transducer and activator of transcription (JAK/STAT) pathway [50], also was induced in response to both stimuli. Of interest, decreased SOCS activity in CD14-deficient murine macrophages has been associated with an exaggerated TLR mediated pro-inflammatory cytokine response to phagocytosed Bb (personal communication from Dr. Tim Sellati).

A surprisingly large number of genes associated with diverse metabolic functions were also contained in this core group. This finding indicates that immune cells have the capacity to down-regulate various genes associated with metabolic functions which are not critically needed during inflammatory responses. Some genes which encode cell surface receptors associated with immune signals were also down-regulated. This was the case for CCR2, which is linked to the cell surface chemokine receptor for MCP-1 [51], and for IFNGR1, which encodes the IFN-γ receptor-1 protein [52]. Of note, mouse macrophages TLR2 stimulation with a synthetic ligand caused a similar decrease in IFNGR-1 transcription [52] Conversely, NALP12 (Monarch1/PYPAF7) which is a negative regulator of TLR-induced inflammatory responses [53] was down-regulated by both stimuli.

### Genes exclusively or more intensely up-regulated by live *Borrelia burgdorferi* (Table 1)

As already noted above, a large number of genes were either exclusively or more intensely up-regulated by live as opposed to lysed Bb. Within this group we found several genes that encode for monocyte-derived chemokines (CCl3L1, CCL3, CCL3L) [54], as well as two monocyte/macrophage-associated growth factors (GMCSF and GCSF) [55]. This group also encompassed a number of genes which are known to be directly or indirectly associated with phagocytosis and/or assembly of phagolysosomes. PI4K2B, a cytosolic phosphoinositol kinase which regulates vesicular trafficking during phagocytosis [56] and LAMP3 (lysosome associated membrane protein 3), a late endosomal maturation marker [57], were both strongly up-regulated. TNF-α and IL-6, which encode for two cytokines previously shown to be secreted in large quantities in response to live Bb in the PBMC stimulation model [32], were also included in this group. In that prior study we also showed that phagocytosed Bb generated signals that activated natural killer (NK)-cells inducing them to produce INF-γ. Herein, the transcript for INF-γ was more than 40-fold higher in PBMCs exposed to live spirochetes. Correspondingly, the genes for CD69 [58] and SLAMF7 (also known as CS1) [59], which are both associated with NK cell activation, also were intensely up-regulated by live bacteria. As a likely consequence from the paracrine effects of INF-γ, STAT1 was robustly up-regulated by live spirochetes. In agreement with this assumption, the gene for NMI (N-myc STAT1 interactor) which is directly involved in STAT-dependent transcription of IFN-γ [60], was exclusively regulated by live Bb. The transcript for FXYD6, which codes for a protein that regulates Na,K-ATPase channels by altering their affinity for Na^+^ and K^+^ ions [61], was up-regulated only by live spirochetes. The latter is particularly noteworthy given that phagocytosed Bb markedly enhanced secretion of active IL-1β by monocytes [32], a process which first requires TLR stimulation trailed by a series of complex signaling events that lead to assembly of the inflammasome, activation of caspase-1 and cytosolic cleavage of pro-IL-1β [62]. Changes in cytosolic ionic composition following phagocytosis, particularly loss of intracellular K^+^, could thus provide the necessary signals required to activate the NALP3 inflammasome [63],[64].

Live spirochetes also more intensely or exclusively regulated several genes associated with cell damage and repair than bacterial lysates. This finding is in accord with our prior demonstration that phagocytosed Bb, but not bacterial lysates, has the capacity to initiate programmed cell death responses in human monocytes [33]. Gene transcripts within this group included PARP9 (Poly ADP-ribose polymerase9); PARP10, PARP12, PARP14 and Galectin9 (LGALS9). The catalytic activity of PARPs has been shown to be stimulated by DNA strand breaks which occur during programmed cell death. Within this cluster, PARP9 and PARP10 were exclusively up-regulated by live Bb. These two PARPs have not been previously associated with apoptosis [65], suggesting that phagocytosis of Bb may initiate novel mechanism of cell damage and repair. Galectin9 is a β-galactosidase binding lectin which is present in activated macrophages and DCs [66], and has the capacity to incite apoptosis via the calcium-calpain-caspase-1 dependent pathway [67].

Canonical TLR1/2 signals are not known to promote transcription of type I interferons [40],[41]. It was, therefore, particularly noteworthy that live Bb induced transcription of IFN-β and several type I interferon-associated genes in human PBMCs (**Table 1**). Within this cluster there were multiple interferon-induced protein transcripts (Mx1, IFIT1, IFIT2, IFIT3, IFI16 and RSD2), as well as several interferon-regulated genes known to code for molecules involved with protein ubiquitination or ubiquitin-like functions (ISG15, HERC5, UBE2L6 and USP18). The transcript for ISG15, which featured prominently within this cluster, codes for a ubiquitin-like molecule that is conjugated to intracellular target proteins after type I interferon stimulation [68]. HERC5 is an IFN-β-inducible E3 protein ligase [69] which localizes to the cytoplasm and perinuclear region of cells and is required for ISGylation of ISG15 [68]. UBE2L6 is a conjugating enzyme also responsible for protein ISGylation, while USP18 is a protease that specifically removes ISG15 [70]. The gene for ISGF3G (also known as IRF9), a translational regulator known to associate with phosphorylated STAT1/2 heterodymers to form a complex termed ISGF3 transcription factor, also was up-regulated by live Bb. ISGF3 enters the cell nucleus and binds to the IFN-stimulated response element (ISRE) to activate the transcription of interferon stimulated genes [71]. IRF7, which was up-regulated by live Bb but not by lysates, is expressed constitutively in monocytes and DCs but can be induced following single-stranded RNA mediated activation of endosomal TLR7 or TLR8 [72]. IRF7 also can be up-regulated by IFN-β through a cell surface initiated paracrine loop that activates STAT1 [73].

### Genes exclusively or more intensely down-regulated by live *Borrelia burgdorferi*

Several genes associated with cell signaling, ion and metal transport systems, and cytoskeleton architecture were more intensely or exclusively down-regulated by live Bb (**Table S2**). The gene for TLR6, which together with TLR1 recognizes diacylated lipoproteins [74], was intensely down-regulated by both stimuli, but much more so by the live spirochetes. Since Bb does not have diacylated lipoproteins on its outer membrane [16], this finding suggests that inflammatory cells are capable of fine-tuning transcriptional responses depending on the biochemical configuration of the lipoproteins encountered. The transcript for CD91 (LRP1), a transmembrane receptor which is known to bind the anti-apoptotic molecule α-2-microglobulin and the heat shock protein gp96 [75], was also in this group. This suggests that in response to live Bb innate immune effector cells shift inflammatory responses away from those that are associated with exogenous damage-associated molecular patterns (DAMPs).

### Genes found to be more strongly or solely up-regulated in response to borrelial lysates

A small group of genes were more intensely or exclusively up-regulated by bacterial lysates (**Table S3**). Of particular interest we found three genes that encode for matrix metallopeptidases (MMP1, MMP10 and MMP19). MMP-9 has been previously shown to be induced in both human and murine monocytic cells in a TLR2 dependent manner [76],[77]. MMP10 was also previously found to be up-regulated in mouse cells infected with live Bb [78]. Several genes associated with structural molecules, including two which are involved in keratin formation (KRT2A and KRTAP17), were exclusively up-regulated by the lysates. Similar structural genes have been shown to be up-regulated in joint tissue obtained from a murine Bb induced arthritis model [79].

### Quantitative Real Time Reverse Transcriptase PCR (RT-PCR) confirmation of select genes differentially regulated in stimulated PBMCs

We previously demonstrated by quantitative real time reverse transcriptase PCR (qRT-PCR) that the transcripts for TNF-α, IL-6 and IFN-γ were more intensely up-regulated by live Bb than bacterial lysates [33]. As shown in **Table 2**, herein we also confirmed by qRT-PCR that live bacteria induced IRF-7, IFN-β, STAT-1, and LGALS9. Although the transcript for galectin-9 was confirmed to be up-regulated by live spirochetes, exclusive down-regulation of this transcript by the lysates as shown in the array could not be corroborated by qRT-PCR. Excluding LGALS9, the live to lysed transcriptional values determined for each individual gene correlated well between the two methods used (R^2^ = 0.99, 95% CI: 0.93–0.99, p<0.001). IL-1β which was intensely up-regulated by live Bb stimulated PBMCs, was not significantly regulated in the PBMC array analysis. On the other hand, as will be discussed below, several type I interferon genes differentially up- or down-regulated in the array were identically regulated in isolated monocytes stimulated under comparable conditions (**Table S4**). Biologic validation for the transcriptional responses denoted in the PBMC array also could be ascertained from prior experiments where we utilized the Bb-PBMC stimulation model [32],[33]. For instance the revelation that Bb induced programmed cell death in monocytes is in accord with the finding herein that several genes associated with apoptosis were up-regulated by live spirochetes. The sizable increase in the transcript for CD69 shown in the array is also consistent with the prior demonstration that NK-cells express this cell surface activation marker when exposed to live Bb [32].

**Table 2:** RT-PCR confirmation of array generated transcriptional responses.

Symbol	PBMC Array *		PBMC RT-PCR **	
	Live Bb	Lysed Bb	Live Bb	Lysed Bb
TNF-α	35.2	3.9	105.2 #	7.2 #
IL-6	1228.7	582.1	852.1 #	234.6 #
IFN-γ	88.3	2.1	1067 #	6.8 #
IRF-7	5.8	1.2	48.1	8.1
INF-β	47.4	4.7	26.7	1.7
STAT1	12.3	3.1	42.7	7.7
LGALS9	3.3	0.1	4.6	1.7
IL-1β	NS	NS	84.7 #	39.5 #

*: Values are based on the normalized gene intensity values between live or lysed Bb vs. unstimulated cells.

**: Values are based on fold increase/decrease in gene transcripts between live or lysed Bb and unstimulated cells.

# These three values are adapted from experiments which were previously reported by Cruz et al. [33].

### Live *Borrelia burgdorferi* enhances pro-inflammatory cytokine transcription and secretion in isolated human monocytes

**Figure 2:**
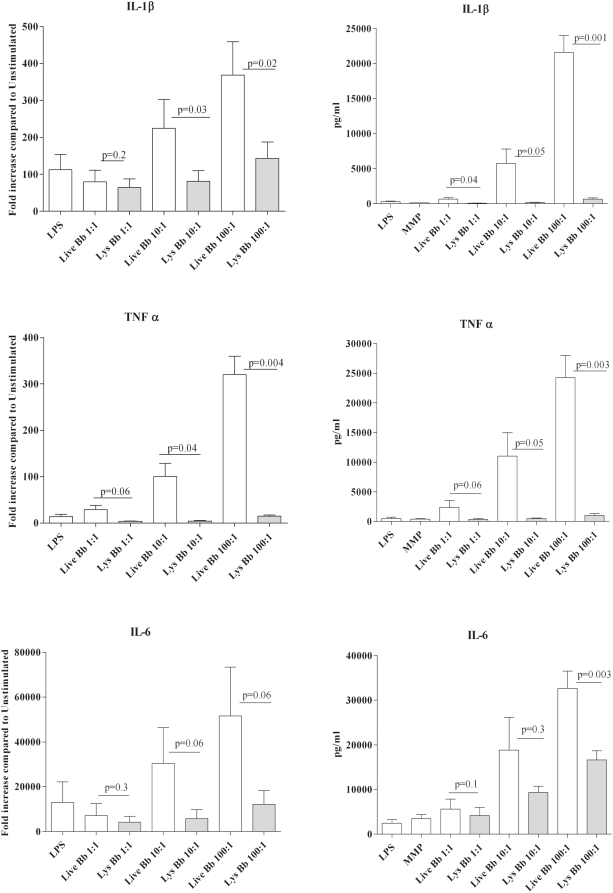
Live *Borrelia burgdorferi* elicits greater cytokine transcription and secretion from isolated human monocytes than borrelial lysates. Monocytes were incubated with live or lysed Bb at various MOIs (1∶10∶100), 100 ng/ml of lipopolysacharide (LPS) and 10 µg/ml the TLR2 synthetic ligand MMP (Mitogenic pentapeptide). Transcription (4 hour stimulation experiments) and secretion (8 hour stimulation experiments) of IL1-β, TNF-α and IL-6 was determined by either qRT-PCR (fold increased in transcript copies compared to unstimulated cells) or bead array cytokine concentrations (pg/ml) in supernatants. Bars depict the means+/−standard error of the mean from a minimum of four independent experiments. P-values for transcriptional and translational comparisons between live and lysed Bb for each of the equivalent MOIs (1, 10 and 100) are shown above the corresponding bar.

**Figure 3:**
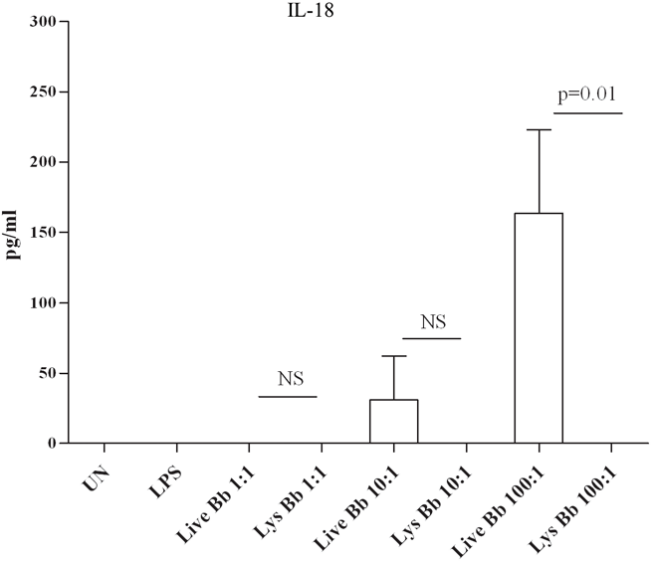
IL-18 is secreted in response to live Bb but not lysates. Monocytes were either unstimulated or incubated with live or lysed Bb at various MOIs (1∶10∶100) and 100 ng/ml lipopolysacharide (LPS). Secreted IL-18 was determined by ELISA as described in the Methods. Bars depict the mean value (pg/ml)+/−standard error of the mean from three independent experiments. P-values calculated for comparative cytokine production between cells stimulated by live and lysed Bb at equivalent MOI are shown above the corresponding bars. NS = not significant.

The transcriptional responses in the PBMC whole genome array, in conjunction with results from our prior *ex vivo* stimulation studies [32],[33], provided substantial evidence that phagocytosis of live Bb triggers inflammatory responses in PBMCs that differ quantitatively and qualitatively from those generated by bacterial lysates at the cell surface. In this study we used the isolated monocytes to verify that this cell was indeed a major source of the transcriptional responses generated by live Bb and that the enhanced responses elicited by viable spirochetes were not dependent on signals or cytokines derived from other mononuclear cells in the PBMC mixture. We first had to verify that uptake of the spirochetes by highly purified human monocytes was equivalent to the uptake previously shown in the PBMC-Bb stimulation model. Not surprisingly, a large percentage of the monocytes (Mean 63%: SE+/−9.6%) cultured with Bb (MOI 100∶1) contained fully or partially degraded fluorescent bacteria (see microscopy section below). Additionally, a similar percentage of CD14+ monocytes were also GFP+ by flow cytometry (data not shown). We then confirmed that live Bb was significantly more potent than borrelial lysates for inducing transcription and secretion of IL-1β, TNF-α and IL-6 (**Fig. 2**). Live spirochetes also induced higher levels of IL-10 (data not shown); indicating that the enhanced response associated with phagocytosed spirochetes also includes the synthesis of anti-inflammatory cytokines which others have shown to be important for control of LD [42],[80],[81]. The marked increases in the amount of secreted IL-1β suggest that phagocytosed live spirochetes induce signals resulting in the activation of caspase-1, which is a known mechanism for cleavage of pro-IL-1β [82]. Additional evidence for caspase-1 activation was obtained by the demonstration that live Bb also induced monocytes to secrete IL-18, which though constitutively expressed also requires proteolytic processing by activated caspase-1 [83]. Of particular importance, neither bacterial lysates nor the TLR-4 ligand LPS were capable of inducing monocytes to secrete IL-18 (**Fig. 3**). Alternatively, IL-1β could be secreted in response to other caspases which are linked to the initiation of apoptosis.

### Live Bb induces Type I interferons in isolated human monocytes

**Figure 4:**
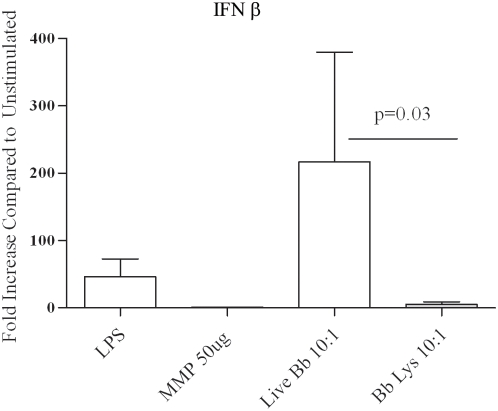
Live Bb, but not borrelial lysates or synthetic TLR2 ligands, induce IFN-β transcription in isolated human monocytes. Monocytes were incubated with live or lysed Bb MOI (10), 100 ng/ml of lipopolysacharide (LPS) and the 10 µg/ml of the TLR2 ligand MMP (Mitogenic pentapeptide). Transcription of IFN-β was determined by qRT-PCR. Bars depict the means+/−standard error of the mean from a minimum of four independent experiments. P value shown corresponds to the statistical comparison between live and lysed Bb (MOI 10∶1) induced IFN-β transcripts.

**Figure 5:**
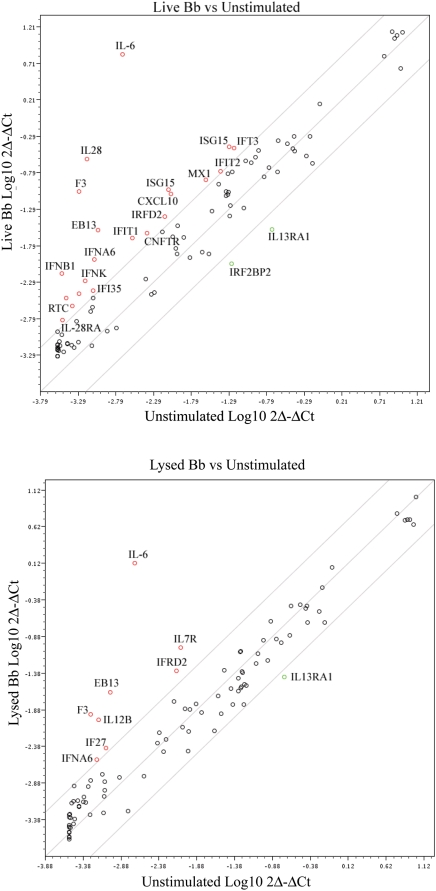
Differentially regulated type I interferons genes monocytes were incubated with live or lysed Bb MOI (10∶1) and gene intensity values were determined by a type I interferon. RT^2^ Profiler Array system. (A) The figure shows comparative analysis between gene transcript values generated by monocytes stimulated with live Bb vs unstimulated cells. Genes depicted outside the lines were 4 fold higher or lower and deemed to be statistically significantly differentially regulated (p<0.01) in the array. (B) Comparative analysis between gene transcript values generated by bacterial lysates vs unstimulated cells.

One of the most significant findings from the PBMC array analysis, and subsequently substantiated by qRT-PCR (**Table 2**), was the discovery that live Bb induced transcription of type I interferons. As shown in **Fig. 4**, live Bb was also capable of inducing a distinct increase in transcription of IFN-β in isolated human monocytes, while a similar response was not observed with borrelial lysates or a high concentration of a synthetic TLR2 ligand (MMP 50 µg/ml). The finding that LPS induced transcription of IFN-β is in line with prior observations demonstrating that TLR4 activation can generate type I interferons through the MyD88-independent TRIF-dependent pathway [84]. To better characterize the breadth of the type I interferon responses generated by live or lysed spirochetes in monocytes, we then utilized a Type I interferon RT^2^ profiler array. This method allows simultaneous, quantitative measurement of 84 Type I interferon associated gene transcripts. Although some overlap did occur in the type I interferon transcriptional responses generated by live and lysed Bb, without exception the response was of greater intensity in monocytes stimulated by live bacteria (**Fig. 5A and 5B** **and** **Table S4**). Live Bb not only exclusively induced transcription of IFN-β, but also transcription of the gene that encodes for IFN-κ, whose functional profile resembles that of IFN-β [**85**]. The gene for the interferon inducible CXCL10 (IP-10), which encodes the ligand for CXCR3, also was significantly up-regulated by live Bb. Increases in CXCL10 mRNA have been shown in dermal lesions [86] and joint fluid [87] of LD patients. Several other type I interferon inducible genes including ISG15, IFIT1, IFIT2 and IFIT3 also were strongly up-regulated by live Bb in monocytes, as in the PBMC system. IL-6 was induced by both live and lysed Bb, which is not surprising since this cytokine is known to be secreted in response to stimuli other than those associated with type I interferons; including TLR2 ligands as well as borrelial lysates [33].

### Isolated monocytes contain phagocytosed and degraded *B burgdorferi* but not intact spirochetes

**Figure 6:**
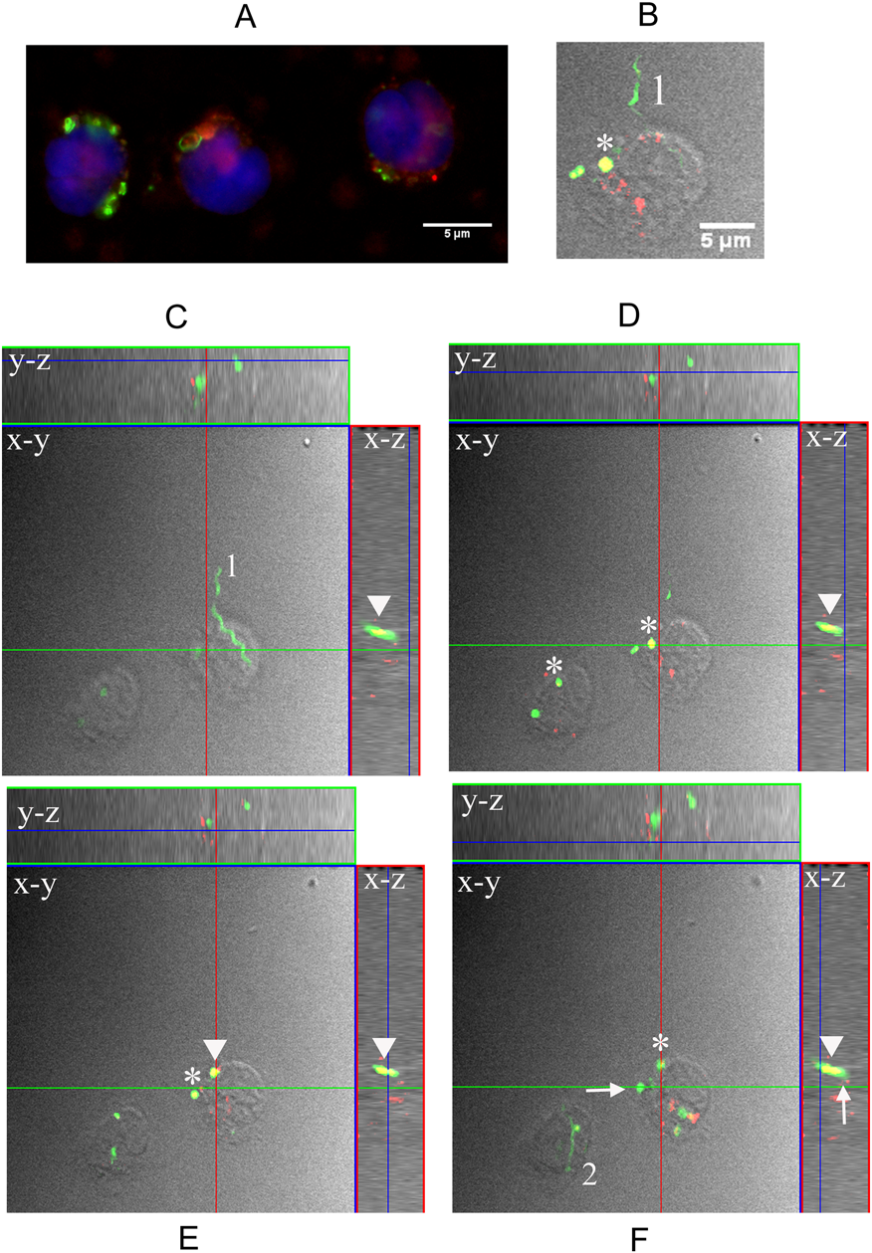
Isolated monocytes contain phagocytosed and degraded *B. burgdorferi* but not intact spirochetes. (A) Epifluorescence image (100×) acquired from human monocytes incubated for 4-hours with Bb-GFP (MOI 100∶1) and labeled with the cell membrane marker FM4-64 (red) and the nuclear dye DAPI (blue). Phagocytosed (both coiled and degraded) Bb-GFP and fragmented nuclei were observed. (B–F) Orthogonal view (y,z and x,z axes) of optical sections (x,y axes) through a confocal stack of isolated monocytes incubated with Bb-GFP (MOI 100∶1) and labeled with lysotracker (red). (B) Digital enlargement of an extracellular spirochete (labeled # 1) in close proximity to a monocyte. Panels C–F are presented in 2 µm increments [(C) 6 µm, (D) 8 µm (E) 10 µm and (F) 12 µm] through the depth of the monocytes (total depth = 14 µm). Arrows and asterisks point to several internalized and degraded Bb-GFP, whereas colocalized phagolysosomes and fluorescent spirochetes shown in yellow are indicated by the arrowhead. The numbers 1 and 2 are placed next to individual extracellular Bb-GFP. Shapes within x,y axes are represented with their corresponding position in the x,z and y,z axes.

The unique transcriptional responses and cytokine outputs demonstrated here highlight two distinct consequences resulting from the interaction of Bb with human phagocytic cells: (1) a markedly intensified TLR mediated pro-and anti-inflammatory cytokine output, (2) activation of signaling pathways generally associated with intracellular bacteria (i.e. *Listeria monocytogenes* and *Franciscella tularensis* [88],[89]) and some extracellular pathogens (i.e. group B streptococcus [90]), which can escape the confines of the phagosome to trigger cytosolic inflammatory responses. Although phagocytic cells have been shown to internalize and degrade Bb [32],[33],[35],[39],[91], consideration of the signaling issues raised above prompted us to visually re-examine the fate of the spirochete when it comes into contact with the monocyte. Epifluorescent microscopy revealed that individual monocytes contained several fluorescent vacuoles with either bacterial coils and/or partially or fully degraded spirochetes (**Fig. 6A**). Of interest, and in concert with our prior demonstration that live Bb induces programmed cell death in monocytes [33], cells also exhibited various stages of nuclear fragmentation (**Fig. 6A**). This finding, which is traditionally associated with apoptosis, correlated with a dose dependent decrease seen in monocyte counts in response to live Bb (data not shown). Because it was not always possible to determine if intact spirochetes were located intra- or extracellularly using epifluorescent images, we then used confocal microscopy which is better suited for such purpose (**Fig. 6B–F**). The representative horizontal optical slices shown in the figure reveal two intact spirochetes that are in close association with the monocyte and several degraded bacteria contained within intracellular vacuoles. Colocalization of GFP fluorescent bodies with Lyso-Tracker dye (red) provided evidence that some of the digested bacteria were inside phagolysosomes. To determine the precise location of intact spirochetes in relation to the intracellular vacuoles, optical slices were then assembled into stacks and cut perpendicularly (y,z axes) and transversally (x,z axes) to the imaged planes generating an orthogonal view of the spirochetes. The resulting side and top views of several hundred reconstructed images allowed us to conclude that intact spirochetes, when present, were not within the cell cytosol.

### Macrophages from TLR2 deficient mice are activated in response to live *B. burgdorferi* but not borrelial lysates

**Figure 7:**
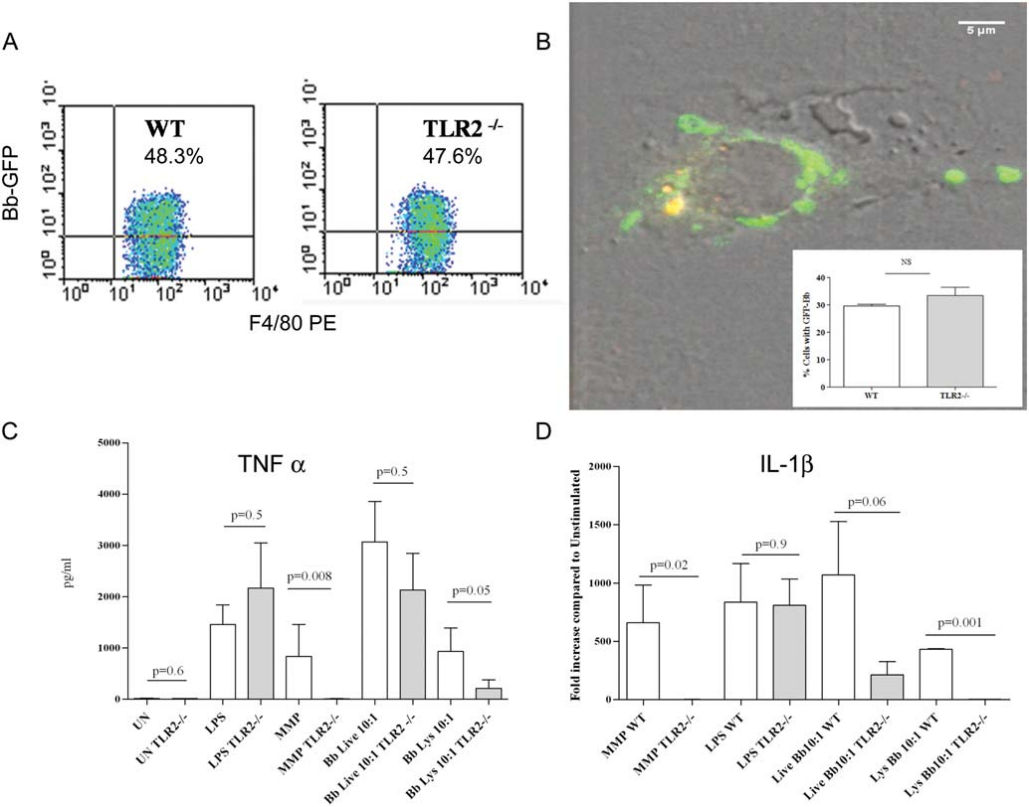
TLR2 deficient and wild type murine macrophages (peritoneal macrophages or bone marrow derived macrophages) internalize *Borrelia burgdorferi* (Bb), and generate similar cytokine responses. (A) Wild type (WT) or TLR2-deficient (TLR2^−/−^) mouse derived peritoneal macrophages were incubated for six hours with Bb-GFP (MOI 100∶1) and analyzed for GFP expression by flow cytometry. Individual macrophage populations shown in the cytograms were selectively gated for analysis based on F4/80 PE expression and Bb-GFP signal. (B) Representative confocal image of internalized Bb-GFP also labeled with lysotracker in TLR2^−/−^ bone marrow derived macrophages incubated for six-hours with live Bb (MOI 100∶1). Inset shows a bar graph depicting the percentage of WT or TLR2^−/−^ macrophages that had spirochetes contained within phagosomal vacuoles. (C) Murine peritoneal macrophages were stimulated for six-hours with: 100 ng/ml of LPS, 10 µg/ml of Mitogenic Pentapeptide (MMP), live Bb-GFP or spirochetal lysates (MOI 10∶1) and compared to unstimulated (UN) cells. The bars represent the average TNF-α concentration (pg/ml) and standard error of the mean calculated from five independent experiments. (D) RNA from similarly stimulated bone marrow derived macrophages (MOI 10∶1) was extracted for qRT-PCR pro-IL-1β quantitation. Fold increase in transcript copies between each of the stimuli is compared to unstimulated cells. Bars depict the means+/−standard error of the mean from a minimum of three independent experiments. P values shown in both C and D correspond to the statistical comparison between WT and TLR2 −/− stimulated macrophages.

**Figure 8:**
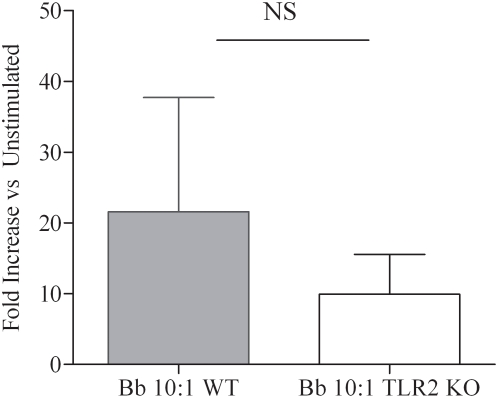
Wild type (WT) and TLR2 deficient bone marrow derived macrophages (BMDMs) generate similar IFN-β responses to Bb. WT or TLR2−/− mouse BMDMs were incubated for six hours with Bb (MOI 10∶1). Fold increase in IFN-β transcript copies measured from Bb stimulated BMDMs are compared to control values from unstimulated cells. Bars depict the means+/−standard error of the mean. Values were not statistically significantly different.

The finding that live Bb generates greater transcription and secretion of pro-inflammatory cytokines than similar amounts of bacterial lysates can have two possible explanations. One is that TLR2 signaling proceeds more efficiently or intensely when spirochetes are phagocytosed. The other is that induction of the cytokine responses resulting from internalization of Bb is not exclusively TLR2-dependent. Both of these possibilities are supported by prior work demonstrating that upon phagocytosis of microbial pathogens or bacterial products, TLRs that are on the cell surface can also be recruited to the phagosome and thus become available for signaling [92],[93]. The most straightforward approach to distinguish between these two possibilities was to examine the cytokine responses to Bb in TLR2^−/−^ murine macrophages. Because the source of the mouse macrophages (bone marrow vs peritoneal derived) has been previously shown to be linked to differing uptake as well as cytokine outputs, in this study we elected to use each cell lines under separate experiments. Consistent with prior observations [31] we demonstrated by using flow cytometry and confocal microscopy that uptake and degradation of spirochetes was not significantly affected despite the absence of TLR2 (**Fig. 7A and 7B**). WT and TLR2^−/−^ macrophages were then stimulated with live or lysed spirochetes (MOI 10∶1) to appraise the output of selected cytokines. Compared to their WT counterparts, macrophages harvested from TLR2-deficient mice and stimulated with live Bb secreted only moderately diminished amounts of TNF-α (∼25% less) (**Fig. 7C**). Decrease responses were slightly more pronounced for IL-6 (1611 pg/ml vs. 604 pg/ml) and IL-10 (248 pg/ml vs. 58 pg/ml). In contrast, at similar MOIs the response to lysates was virtually eliminated in TLR2^−/−^ cells (**Fig. 7D**). While bacterial sonicates have numerous structural components available for cell signaling, the severely impaired cytokine output in TLR2^−/−^ macrophages indicates that the response to the lysates is principally due to spirochetal lipoproteins. Although transcription of IL-1β in TLR2^−/−^ macrophages stimulated with live Bb was not as robust as compared to the WT cells, the absence of TLR2 once again did not eliminate the response. Whereas induction of IL-1β in TLR2^−/−^ cells was absent upon stimulation with bacterial lysates. Unlike human monocytes which were capable of secreting large quantities of IL-1β in response to live bacteria, neither WT nor TLR2 deficient murine macrophages were able to secrete detectable amounts of this cytokine (data not shown). On the other hand, phagocytosed live Bb (MOI 10∶1) was able to similarly induce transcription of IFN-β in both WT and TLR2 deficient bone marrow derived macrophages (**Fig. 8**).

### Proinflammatory cytokines responses to Bb are not TLR5-dependent

**Figure 9:**
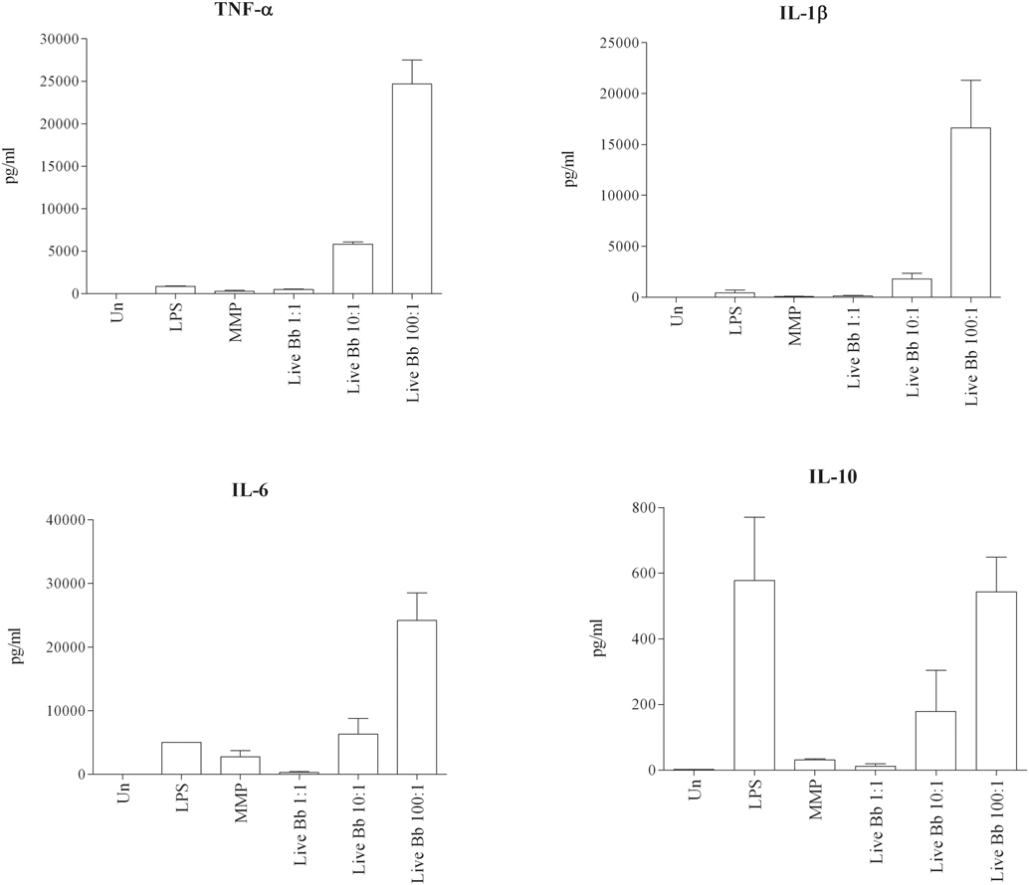
Flagellin deficient Bb induces NF-κB mediated cytokine responses in human monocytes. Isolated human monocytes were incubated with live flagellin deficient Bb at various MOIs (1∶10∶100), 100 ng/ml of lipopolysacharide (LPS) and 10 µg/ml of the synthetic TLR2 ligand MMP (Mitogenic pentapeptide). IL-1β, TNF-α, IL-6 and IL-10 protein concentrations (pg/ml) were quantitated (see Methods) in supernatants obtained from 8 hour stimulation experiments. Bars depict the means (pg/ml)+/−standard error of the mean for each cytokine measured from two independent experiments.

TLR5 can be expressed in endosomal structures [94],[95] and can be activated by bacterial flagellin to induce synthesis of pro-inflammatory cytokines [96]. Although not exposed on *B burgdorferi's* outer membrane, flagellin is a major constituent of the spirochete's periplasmic flagellar structures [97] and thus can become accessible for endosomal TLR5 signaling following degradation of the spirochete within phagolysosomal vacuoles. In concert with this premise, two previous animal studies provided evidence that TLR5 may be partially responsible in generating pro-inflammatory responses to phagocytosed Bb [31],[95]. Thus to examine the contribution of TLR5 ligation with TLR2 mediated signals, herein we measured human monocyte cytokine output in response to a Bb strain known to be deficient in flagellin [98]. Despite the elongated structure, the mutant spirochetes were readily phagocytosed and degraded within phagolysosomes (data not shown). The flagellin deficient spirochetes were also capable of inducing human monocytes to secrete very similar levels of IL1-β, TNF-α, IL-6 and IL-10 (**Fig. 9**), than elicited by wild type Bb (**Fig. 2**). This finding indicates that when TLR2 is available for signaling, TLR5 does not appear a play a significant role in production of cytokines in response to phagocytosed live Bb.

## Discussion

In the course of natural infection with the extracellular pathogen *B. burgdorferi* phagocytic cells are considered to be the first-line of host defense against the bacterium [2],[25],[32],[33],[99]. Immune cell activation by the spirochete has generally been ascribed to outer membrane lipoprotein-TLR1/2 mediated inflammatory responses [8]–[15]. Evidence ascertained herein from the PBMC array, and subsequently corroborated using similarly stimulated monocytes, corroborated the important contribution of Bb-cell surface TLR1/2 mediated activation in response to the spirochete. Our study results also make obvious that a far more intense and diversified innate immune response coincides transcriptionally and microscopically with phagocytosis and degradation of live spirochetes and maturation of the phagosome. Most prominently, the innate immune signals generated by phagocytosed live Bb led to an enhanced TLR-mediated pro- and anti-inflammatory cytokine output, secretion of active IL-1β and IL-18, as well as induction of type I interferons.

TLRs continuously sample the extracellular environment and inform the cell to react to PRRs by facilitating cellular responses via inflammatory pathways which culminate in cytokine production and cell activation [11],[41]. Human macrophages, which originate as monocytes in the peripheral blood, express a substantial complement of both cell surface as well as endosomal TLRs [100]. These cells thus have the capacity to sense *B. burgdorferi's* extensive lattice of outer membrane lipoproteins [23],[101]. Although the stimulation experiments were not done under conditions designed to prevent uptake of live spirochetes or bacterial components, the transcriptional responses elicited by both live and lysed Bb in PBMCs, for the most part were representative of cell surface TLR1/2 mediated activation. These responses were perhaps best exemplified by the differential regulation of IL-8 as well as several other chemokines. IL-8 is known to be secreted in response to purified spirochetal lipoproteins [45],[46] as well as Bb lysates [47]. Surface signals were also capable of up-regulating several genes associated with molecules that regulate TLR responses, including PI3K [102],[103] and SOCS3 [50]. Interestingly, PI3K is also associated with non-opsonic phagocytosis [102] and thus may play an important role facilitating spirochetal binding to the bacterium's putative cell surface phagocytic receptor. Overall, these core responses suggest that TLR-cell surface activation, probably in concert with the engagement of the spirochetes putative phagocytic receptor, set the stage for the more intense TLR-dependent and -independent responses generated by phagocytosed spirochetes.

The enhanced TLR-mediated cytokine production could be the result of several nonexclusive mechanisms broadly divided into three categories; (1) a more efficient activation of recruited cell surface and endosomal TLR1/2 receptors by spirochetal lipoproteins, (2) engagement of additional endosomal TLRs by internalized and degraded spirochetes, and (3) cooperation between multiple TLR receptors from within the phagosomal vacuole. Although TLR-PAMP interactions were originally studied as cell surface phenomena; it is now well documented that surface TLRs, including TLR2 and TLR5, can be recruited to endosomal membranes where they become available for signaling [79],[92],[93],[95],[104]. The visualization of bacterial coils contained within phagosomal vacuoles provided a snapshot for the intimate physical interactions that very likely take place between the spirochete and vacuolar structures (**Fig. 6A**). The very close proximity between spirochetal lipoproteins and recruited TLRs can be envisioned as a mechanism that facilitates activation of TLR receptors. Following degradation of the bacterium, liberated lipoproteins then would also be available to more efficiently engage of their cognate TLR receptors. Because TLR2 did not appear to be necessary for phagocytosis, or critically required for cytokine secretion in response to live spirochetes, we propose that other TLRs are involved in generating these responses. Two previous studies, one using stimulated murine macrophages and RAW cells, and the other rhesus microglia, demonstrated that TLR5 signals do contribute to cytokine production in response to phagocytosed Bb [31],[95]. Our demonstration that the *flaA* mutant Bb induced similar levels of TNF-α, IL-1β, IL-6 and IL-10 in human monocytes, compared to its wild type counterpart, suggests that as long as TLR2 is available for signaling, TLR5 is not necessary for enhanced cytokine production. Consistent with this theory, silencing of *TLR5* in a recent study had no effect on the production of TNF-α, IL-8, or IL-6 by a monocytic cell line stimulated with live Bb [105]. TLR7, TLR8 and TLR9, all of which are known to be expressed in endosomal membranes [106], may also play an important role in generating the enhanced cytokine responses to internalized Bb. In the end, spirochetes almost certainly engage multiple TLRs concurrently from within the phagosomal vacuole, a conjecture that has been previously demonstrated to occur in response to other bacterial infections [107].

Particularly important for the development of the concept of phagosomal signaling was the markedly enhanced secretion of IL-1β and IL-18 in response to live Bb. Unlike other pro-inflammatory cytokines (i.e. TNF-α), which are linearly induced by TLR activation, the production of biologically active IL-1β requires the integration of NF-*κ*B-mediated transcription of pro-IL-1β followed by activated caspase-1 cleavage of the inactive cytokine [62]. Unlike pro-IL-1β, pro-IL-18 is constitutively expressed in resting monocytes and macrophages [83]; however, like pro-IL-1β it also requires processing into its active form by activated caspase-1. Caspase-1 is activated within a multiprotein complex called the inflammasome [89],[108] in response to a diverse stimuli including intracellular bacteria [89], uric acid crystals [108], toxins, and changes in the ionic composition of the cell [63],[64]. Stimulation of cell surface purinergic receptors by exogenous ATP, following human monocyte activation, is a known mechanism by which bacterial pathogens can lead to cleavage of caspase-1 [64]. Released ATP engages the cell's purinergic ion channel receptors (P2X7) inciting release of intracellular K^+^ which in turn generates signals that lead to assembly of the inflammasome and activation of caspase-1. Consistent with this theory, the transcript for FXYD6, which encodes for a protein that regulates Na^+^,K^+^-ATPase channels by altering their affinity for both ions [109], was exclusively up-regulated in PBMCs stimulated by live spirochetes (see **Table 1**). In similarly stimulated mouse macrophages Bb also induced far greater transcription of IL-1β than bacterial lysates; however unlike human monocytes, murine macrophages were unable to secrete the active cytokine. The disparity in IL-1β secretion between mouse and human cells is due to the inability of murine macrophages to produce endogenous ATP following their activation [110]. These differences not only highlight the importance of studying inflammatory responses in human cells, but also provide indirect evidence for the potential role of P2X7 mediated activation of the inflammasome by phagocytosed Bb. Alternatively, caspase-1 could be activated in response to bacterial flagellin leaking from the phagosome into the cell cytosol. Two prior studies demonstrated that flagellin deficient *Salmonella typhimurium* [111] and *Legionella pneumophila* [112] failed to activate the inflammasome, thus providing clear evidence that flagellin needs to gain access to the cytosol in order to directly activate assembly of the inflammasome. This mechanism is unlikely to be responsible for activation of caspase-1 in the case of Bb, first and foremost because the spirochete does not have the required cellular machinery to secrete noxious molecules into its surrounding environment. Furthermore, the high concentrations of secreted IL-1β in response to flagellin deficient Bb provides further proof that this molecule is unlikely to be directly responsible for activation of caspase-1. In other models activation of caspase-1 was achieved by intracellular bacteria that have the ability to escape unscathed from the phagosome into the cytosolic compartment to directly engage cytosolic receptors and activate the inflammasome [113],[114]. Although the translocation of small amounts of spirochetal components cannot be ruled out in the current study, the evidence presented here demonstrates that intact Bb remains enclosed within phagocytic vacuoles. Interestingly, secretion of IL-1β was greatly impaired in human monocytes infected with a Francisella strain when its natural ability to escape the phagosome was blocked experimentally [115]. Why Bb generates signals that lead to activation of caspase-1 in human monocytes from within the phagosome, while other pathogens do not, requires further analysis.

A principal and novel finding in our study was that type I interferons were differentially regulated in both PBMCs and isolated human monocytes stimulated with live Bb. Type I interferon associated genes were previously shown to be strongly up-regulated in joint tissues of Bb infected mice [43],[79]. The same group provided experimental evidence that type I interferons probably play a very important role in the development of arthritis [43]. These responses are of particular relevance given that lipoprotein mediated TLR2-derived signals do not induce type I interferons [40],[41]. Several other TLRs, including TLR7, TLR8 ad TLR9, are able to launch distinct signaling pathways from within phagosomal vacuoles that differentially regulate type I interferons [116]. Intracellular bacterial deoxycytidylate-phosphate-deoxyguanylate (CpG)-DNA can induce type I interferon transcription through TLR9 [117]; however TLR9 is only expressed at very low levels in human monocytes [106]. Both TLR7 and TLR8 detect ssRNA [100] and thus could also explain the type I interferon responses to Bb upon release of bacterial RNA from degraded spirochetes in the phagosomal vacuole. Human TLR7 is predominantly expressed in lung, placenta and spleen, whereas TLR8 is more abundant in peripheral blood leukocytes, including monocytes [100]. Endosomal TLR8 activates IRF7 via MyD88-dependent^−^pathways involving IRAK1/4 and TRAF6 [72]. Although IRF7 was strongly up-regulated by live Bb in the PBMC array, we were unable to confirm a similar up-regulation of this interferon regulator in human monocytes (data not shown). The latter result could be an indication that in the PBMC model dendritic cells are the principal source of IRF7. Of note, transcription of type I interferon associated genes in response to Bb was recently found to be MYD88 independent in stimulated murine derived bone marrow derived macrophages [43]. Whether or not transcription of type I interferons in response to Bb is MYD88 dependent in human cells has not yet been fully characterized.

Type I IFNs had generally been associated with antiviral immune responses. More recently, an increasing body of evidence points out that induction of these cytokines also occurs in response to infection with both intracellular [88],[118],[119] and extracellular bacteria [90],[120]. IFN-inducing bacterial ligands are primarily detected, with few exceptions, following entry of the bacterium into the cell cytosol. For the most part, the specific cytosolic receptors activated to generate the type I interferons are not known. The intracellular bacterium *L. monocytogenes* induces type I interferons through an MYD88-independent pathway [88],[116], and to generate this response it requires Listeriolysin O (LLO) mediated escape from the phagosome into the cell cytosol [116],[121]. Neither the ligand presented by cytoplasmic LM nor the receptor associated with the type I interferon response has been fully characterized. *Streptococcus agalactiae* (GBS), an extracellular pathogen associated with severe perinatal infections, also induces type I interferons in human monocytes [90]. However, unlike Bb, GBS can generate toxins that affect the integrity of the phagosome allowing bacterial components to escape into the cytosol to engage cytosolic receptors. Released GBS DNA can activate the serine-threonine kinase TBK1 causing phosphorylation of IRF3 and induction of IFN-β. In the case of *Streptococcus pyogenes* (GAS), induction of type I interferons was shown to be MYD88-independent [120]; and like Bb, GAS did not require escape into the cytosol to generate this response. Whether or not Bb induced type I interferon responses are initiated from within the phagosome, or by signals generated by released bacterial products into the cell cytosol is not known at this time. It is also not known if Bb initiated programmed cell death responses have a role in generating type I interferons through cross priming of cytosolic receptors.

Collectively our results highlight the ability of phagocytosed Bb to induce diverse and more intense innate immune signals which are mechanistically distinct from those generated when spirochetal lipoproteins engage cell surface PRRs. They also demonstrate that human monocytes are a major source of the transcriptional responses generated by live Bb in a mixed cell system (PBMCs), and that these responses are not dependent on inflammatory signals or cytokines derived from other immune cells. It is our contention that the phagosomal signals generated in response to live Bb allow the host to control the spirochete through a number of non-exclusive pathways, that are both TLR2 dependent and independent, and include a type I interferon response. Whether or not the type I IFN response is favorable to the human host, or detrimental as in the mouse arthritis model, remains unknown and deserves additional study.

## Methods

### Human subjects

Eligible participants were healthy volunteers of either sex, between 18 and 60 years of age, and without a clinical or prior history of reactive laboratory tests for LD. After obtaining written informed consent, blood was collected by the University of Connecticut Health Center's (UCHC), General Clinical Research Center (GCRC) personnel using standard venipuncture techniques. Volunteers were confirmed to be sero-negative for LD by standard serological tests performed by the UCHC clinical laboratory. Individuals were considered ineligible if they had underlying chronic diseases, were acutely ill, and/or were taking anti-inflammatory medications or any form of immunosuppressive agents. All procedures involving human subjects were approved by the Institutional Review Board at UCHC.

### Bacterial strains

Low-passage Bb 297 transformed with a shuttle vector harboring the gene for green fluorescent protein (GFP) constitutively expressed from *flaB* promoter [122], was propagated in commercially available Barbour-Stoenner-Kelly (BSK) complete medium containing 6% rabbit serum (Sigma-Aldrich, St. Louis, MO) which is certified to be endotoxin free. Spirochetes grown at 33°C were temperature-shifted to 37°C prior to use; organisms were harvested at mid- to late-log phase (4–8×10^7^/ml) by centrifugation at 8,000×g, washed twice in CMRL (Invitrogen, Carlsbad, CA), and resuspended in RPMI medium (Invitrogen). A high passage strain B31 *flaB* mutant (1571), which completely lacks the filamentous portion of the flagellar apparatus (44), was grown and harvested in similar fashion. Spirochetal lysates were prepared by sonicating live organisms for 40 seconds using four separate 10-s bursts with a 550-Sonic Dismembrator (Fisher Scientific, Pittsburgh, PA). The equivalence of live and lysed spirochetes was confirmed for each experiment by SDS-PAGE and silver staining.

### PBMC global gene expression microarray analysis

Freshly isolated PBMCs were obtained by Ficoll density gradient centrifugation from three healthy volunteers as previously described [33]. PBMCs were then incubated 4 hours at 37°C/5% CO_2_ with either live or lysed Bb 297 at an MOI of 10, with 1 µm Fluoresbrite carboxylated polystyrene microspheres (Polysciences, Inc., Warrington, PA) at a bead-to-cell ratio of 10, or without a stimulant. At the conclusion of the incubation period, cells were harvested and RNA was extracted using TRIzol according to the instructions of the manufacturer (Invitrogen). RNA samples were then submitted to the Translational Genomics Core facility at UCHC for gene expression profiling using the Illumina Sentrix Human-6 Expression BeadChip microarray system (Illumina Inc., San Diego, CA). A total of 500 ng of RNA per stimulation condition was converted into complimentary DNA (cDNA) and labeled according to the manufacturer's instructions and then assayed in triplicate for each stimulation condition. For statistical analysis, raw gene intensity values generated for each transcript were background-corrected and normalized using the Qspline option in the Beadstudio software (Illumina Inc.). Statistical analysis was conducted using the R/MAANOVA open source software (Version 1.4) as part of the Bioconductor and R language open source software library (version 2.4.0). The ANOVA analysis was carried out with a fixed effect permutation ANOVA model consisting of the independent variable treatment (live Bb versus lysed Bb). To identify differentially regulated genes, we performed the F-tests F1, F2, F3 and Fs that are implemented in the R/MAANOVA software and assessed gene-centric (F1-test) and array centric (F3 test) variance through comparison of the respective lists of regulated genes with those provided by the F2 and Fs-tests which interpolate between gene centric and array centric variance. R/MAANOVA uses the Benjamini-Hochberg test [123] to perform the false discovery rate, which was set to a p value of <0.005, and pooled across all gene list to create an inclusive master list and selected the subset of highly significantly differentially regulated genes through filtering for genes that demonstrated a change in their expression level of at least 1.3 fold. The differentially regulated genes were organized into clusters using the hierarchical clustering module of D-Chip software (revised 2006) [124] for both genes and samples with the clustering parameter set to (a) Euclidian distance and (b) a p-value of 0.05. Differentially expressed genes were annotated using all publicly available databases including the GenBank, GO and IHOP databases.

### Monocyte isolation and stimulation

Isolated PBMCs were washed 3 times with phosphate buffered saline (PBS), using low speed spins to avoid platelet contamination, then counted using a hemocytometer and resuspended in 30 µl of ice-cold sorting buffer (PBS, 2 mM EDTA, 0.5% BSA, pH 7.2) per 1×10e^7^ total cells. Monocytes were isolated from the pelleted PBMCs using a magnetic cell sorting monocyte isolation kit (Miltenyi Biotech, Auburn, CA). PBMCs were first incubated with metallic bead-conjugated antibodies specific for CD3, CD19, CD123 and CD56. Antibody labeled PBMCs were then passed through two separate ferromagnetic columns. The collected flow-through contained CD14^+^ monocytes (average purity 96.7%), which then were counted, spun and resuspended at 1×10e^6^ per ml in RPMI (Invitrogen) containing 10% fetal bovine serum (FBS). Monocytes were plated and incubated for either 4 or 8 hours at 37°C/5% CO_2_ with live GFP-Bb or equal amounts of lysed spirochetes at multiplicities of infection (MOIs) of 1, 10, and 100 (except were noted in the text), 100 ng/ml of LPS (Sigma-Aldrich), or 10 µg/ml of Mitogenic Pentapeptide (MMP), a synthetic lipohexapeptide corresponding to the N-terminus of *Escherichia coli* murine lipoprotein (Bachem Bioscience, King of Prussia, PA). At the end of the incubation period, culture supernatants were collected and store at −70°C for later cytokine analysis. Monocytes also were harvested for flow cytometry, confocal microscopy and/or RNA extraction depending on the experiment. All culture media and reagents utilized in the stimulation experiments were confirmed to be essentially free of LPS contamination (<10 pg/ml) by Limulus amoebocyte lysate assay quantification (Cambrex, MA).

### Quantitative Real Time Reverse Transcriptase PCR (qRT-PCR)

Up- or down-regulation of selected transcripts generated from the PBMC array was also verified in cDNA from stimulated PBMCs and isolated human monocytes for selected genes by qRT-PCR analysis. RNA was extracted from both stimulated and unstimulated cells using the Paxgene blood RNA kit (Qiagen, Valencia, CA). The quality of the RNA was verified both with the DU 530, Life Science spectrophotometer (Beckman, Fullerton, CA) and Agillent bioanalyzer. Complementary DNA was prepared from extracted RNA samples using a high capacity cDNA RT kit. (Qiagen, Foster City, CA). PCR amplification was performed using 25-µl reaction mixtures which contained 2.5 µl of cDNA, 12.5 µl of universal master mix (Applied Biosystems), 8.75 µl of water and 1.25 µl of each primer probe of interest (20×). Commercially available gene expression assays (Applied Biosystems) were used for amplification of the following transcripts; TNF-α (Hs00174128_m1), IL-1β (Hs00174097_m1), IL-6 (Hs00985639_m1), IL10 (Hs00174086_m1Hs), IRF-7 (Hs00185375_m1); IFN-β (Hs00277188_s1), STAT1 (Hs01014002_m1), USP18 (Hs00276441_m1), LGALS9 (Hs00371321_m1), and FXYD6 (Hs01121135_m1). Internal standard curves for each gene were generated using equal volumes of reverse-transcribed quantitative PCR human reference total RNA (Clonetech, Mountain View, CA) diluted 10-fold to obtain concentrations ranging from 125 to 0.125 ng/µl. Quantitative RT-PCR gene expression assays for the house keeping gene, glyceraldehyde-3-phosphate dehydrogenase (GAPDH) (Hs99999905_m1), were performed using identical aliquots of each cDNA as normalization controls. All amplification reactions were performed in triplicate; control reactions without reverse transcriptase also were performed to confirm the absence of contaminating DNA. Amplification reactions were performed with the iCyclerIQ thermal cycler (Bio-Rad) using the following conditions: 95°C for 10 min, and 40 cycles of 95°C for 15 s and 60°C for 1 min. Cycle threshold (Ct) values for each gene were determined from the linear region of the corresponding amplification plots using software supplied by the manufacturer (Bio-Rad). Expression levels of all transcripts studied were normalized to the GAPDH level and the relative changes in gene expression generated were calculated using the 2^−ΔΔCt^ method [125].

### Isolated monocyte quantitative real-time targeted array analysis for type I interferons

A 102 µl aliquot of cDNA obtained from unstimulated or stimulated monocytes (live or lysed Bb at an MOI of 10∶1) served as the template for qRT-PCR analysis using a human type I interferon signaling pathway array (Superarray Bioscience, Frederick MD). These arrays contain primer pairs for 84 genes implicated in type I interferon signal transduction as well as housekeeping genes and controls in 96-well microtiter plates. This qRT-PCR methodology directly quantifies transcript levels based upon the 2^−ΔΔCT^ [125] method through measurement of SYBR green fluorescence using nan iQ5 real-time PCR detection system (Bio-Rad, Hercules, CA).

### Flow cytometry techniques

#### Human monocyte purity evaluation by flow cytometry

Antibody conjugates were purchased from BD Biosciences Immunocytometry Systems (BDIS) (San Jose, CA) or e-Biosciences (San Diego, CA). Monocytes were harvested from tissue culture plates and washed once in fluorescence-activated cell sorting (FACS) buffer (PBS, 0.1% BSA, 0.055% NaN_3_) in preparation for flow cytometry. Cells were incubated for 10 minutes at 4°C with 10 µg of purified human IgG (Sigma-Aldrich) for Fc receptor (FcR) blocking followed by 20 minute incubation with fluorochrome-conjugated antibodies. After a final wash in FACS buffer, cells were fixed in FACS buffer + 1% paraformaldehyde. Flow cytometry was performed using a FACSCalibur dual-laser flow cytometer (BD); a minimum of 50,000 events were collected for analysis. Monocyte purity was determined by flow cytometry based on CD14 expression and lack of expression of CD3 and/or CD19. Multiparameter files were analyzed using WinMDI v2.8 software (Joseph Trotter, Scripps Clinic). Individual monocyte populations were also selectively gated for analysis based on CD14 expression or Bb-GFP signal.

#### Flow cytometric assessment of spirochete uptake

Mouse peritoneal macrophages (MPMs) were aliquoted into 5 ml Polystyrene round-bottom tubes (BD Biosciences, Bedford, MA) at a concentration of 5×10^5^ cells/600 µl/tube, centrifuged at 250×*g* for 10 minutes followed by 6 hour incubation with GFP-Bb at an MOI of 10. Cells and bacteria were coincubated for 6 hours and then washed twice in FA buffer (BD Microbiological Systems, Sparks, MD) and were fixed in FA buffer containing 1% paraformaldehyde. Sample data was acquired on FACSCalibur dual-laser flow cytometer (BD) and results were analyzed using using WinMDI v2.8 software (Joseph Trotter, Scripps Clinic).

### Cytokine analysis

The Cytokine Bead Array kit (BD Biosciences, San Diego, CA) was used as previously described [2] for simultaneous measurement of tumor necrosis factor alpha (TNF-α), interleukin 6 (IL-6), IL-10, IL-1β, and IL-12 in supernatants from stimulated and control human monocytes. A similar kit was used to measure TNF-α, IL-6 and IL-10 in stimulated mouse macrophages. IL-18 was measured by ELISA using a commercial kit (Bender Med Systems Inc, Burlington, CA).

### Microscopy

Isolated human monocytes or mouse macrophages (where indicated) were incubated with 75 nmol/ml LysoTracker Red (Invitrogen) plus live Bb-GFP or lysates for a total of four hours (human monocytes) or six hours (mouse macrophages). For membrane labeling, isolated cells plus live Bb-GFP were incubated with 5 µg/ml FM4-64 (Invitrogen) for 10 minutes on ice. Stimulated cells were fixed in 1% paraformaldehyde, briefly dried onto slides and mounted in Vectashield containing DAPI (Invitrogen). Cells were visualized using epifluorescence or confocal microscopy. Fluorescent images were acquired on an epifluorescent Olympus BX-41 microscope using a 100× (1.4NA) oil immersion objective equipped with a Retiga Exi CCD camera (Q Imaging, Tucson, AZ) and the following Omega filter sets: DAPI, FITC, and Rhodamine. Confocal images were acquired using a LSM-510 confocal microscope (Zeiss, Oberkochen, Germany) equipped with argon and HeNe lasers. Images were acquired using a 63× (1.3 NA) oil immersion objective (512×512 pixel resolution) at 1 µm intervals. Image processing and analysis were performed using ImageJ (NIH, v1.41b) and LSM Image Browser (Zeiss, v4.2.0.121). The percentage of monocytes or murine macrophages containing intact or degraded fluorescent spirochetes was systematically quantified in an equivalent number of monocytes for each of the conditions studied.

### Mouse macrophage isolation and stimulation experiments

All animal work was approved by the UCHC animal care committee and the research conducted according to institutional guidelines. C57Bl/6 mice deficient in TLR2 were kindly provided by Timothy Sellati, PhD., Albany Medical School. Sex and age-matched wild type (WT) C57Bl/6 mice were purchased from Harlan Laboratories. Experiments were done using either mouse peritoneal macrophages (MPMs) or bone marrow derived macrophages (BMDMs).

#### Isolation of MPMs

Cohorts of TLR2 knockout and WT mice were injected intraperitoneally with 1 ml of 15% Proteose Peptone (BD) three days prior to sacrifice and collection of peritoneal exudate cells by lavage with sterile PBS. Two 5 ml lavages were performed per mouse and kept on ice to prevent adherence to polypropylene tubes. The collections from each mouse were checked for the absence of coliform bacteria by microscopy and clean harvests from the same mouse phenotype were pooled prior to cell counting on a hemocytometer. Pooled cells were washed and suspended in RPMI plus Penn/Strep, plated at 1×10^6^/ml in 24 well plates, and incubated overnight. The wells were washed with warm RPMI to remove non-adherent cells and antibiotics, then replenished with fresh RPMI/10% FBS (1 ml per well). This adherent cell fraction was used in 6 hour stimulation experiments as described below.

#### Isolation of BMDMs

Bone marrow cells from C57Bl/6 mice deficient in TLR2 as well as WT mice were recovered by flushing femurs and tibias with DMEM and then incubated in tissue culture-treated 25 cm^2^-flasks (BD Falcon, BD Biosciences, San Jose, CA) overnight at 37°C with 5% CO_2_ to eliminate adherent fibroblasts, granulocytes, and any contaminating macrophages. The following day, 1×10^7^ suspension cells were maintained in 10-cm^2^ bacteriological Petri dishes (BD-Falcon) for three days with DMEM supplemented with 10% FBS, 20% L292-cell conditioned media, 0.01% HEPES, 0.01% sodium pyruvate, and 0.01% L-glutamine. Cultures were supplemented with five ml of the above-described medium and seven days after isolation, cell monolayers were exposed to ice-cold PBS and were recovered by scraping to be used in stimulation experiments. Single cell macrophage suspensions (MPMs or BMDMs) were seeded into 6-well tissue culture-treated plates at a concentration of 1×10^6^ cells/2 ml/well and allowed to adhere overnight. The following day old media was replaced with the supplemented DMEM media and then spirochetes were added at an MOI of 10 and co-incubated for 61 hours at 37°C in 5% CO_2_. TLR2^−/−^ and WT macrophages were then stimulated with live Bb at MOIs of 10, LPS (100 ng/ml) and MMP (10 µg/ml). MPMs were also analyzed by flow cytometry to detect uptake of fluorescent Bb as described above. Supernatants were assayed for cytokine production using a mouse Cytokine Bead Array kit. RNA was extracted after the six hour incubation period from both stimulated and unstimulated BMDMs using a commercial extraction kit. RNA was then reverse transcribed into cDNA for qRT-PCR analysis. Commercially available mouse gene expression assays (Applied Biosystems) were used for amplification of IL-1β (Mm01336189_m1), TNF-α (Mm00443258_m1) and IFN-β (Mm00439546_s1). Expression levels for both transcripts were normalized to the GAPDH (Mm99999915_g1) level and the relative changes in gene expression generated were calculated using the 2^−ΔΔCt^ method [125].

### Statistical methods

General statistical analysis was performed using GraphPad Prism 4.0 (GraphPad Software, San Diego, CA). Fold increase or decrease for each specific gene transcript assayed by qRT-PCR and cytokine concentrations were compared amongst the different stimulus by using either a paired or unpaired Student *t* test or the equivalent non-parametric methods (i.e. Wilcoxon). For each experiment, both the standard deviation and the standard error of the mean were calculated. *p* values of <0.05 were considered significant.

## Supporting Information

Table S1 (A) Genes similarly up-regulated by live and lysed *B. burgdorferi* (Bb). (B) Genes similarly down-regulated by live and lysed *B. burgdorferi* (Bb).
(0.08 MB PDF)

Table S2 Genes classified as being down-regulated in peripheral blood mononuclear cells (PBMCs) stimulated with live *Borrelia burgdorferi* (Bb) MOI (10∶1), in comparison to cells stimulated with similar concentrations of borrelial lysates.
(0.10 MB PDF)

Table S3 Genes more intensely or exclusively up-regulated by lysed *B. burgdorferi*.
(0.06 MB PDF)

Table S4 Differentially regulated type I interferon associated genes.
(0.07 MB PDF)
