# Supplementary material for: Activation of Human Monocytes by Live Borrelia burgdorferi Generates TLR2-Dependent and -Independent Responses Which Include Induction of IFN-β
Source: PLoS Pathog. 2009 May 22;5(5):e1000444. doi: 10.1371/journal.ppat.1000444 (PMC2679197; doi:10.1371/journal.ppat.1000444)
Supplement: Table S1 — (A) Genes similarly up-regulated by live and lysed B. burgdorferi (Bb). (B) Genes similarly down-regulated by live and lysed B. burgdorferi (Bb). (0.08 MB PDF) [file ppat.1000444.s001.pdf]

Supplemental Table 1A: Genes similarly up-regulated by live and lysed *B. burgdorferi* (Bb)

Transcript	Symbol	Beads/Untreated	Live/Untreated	Lysate/Untreated	Live/Lysate
NM_002852.2	PTX3	0.25	38.16	25.53	1.49
NM_005064.3	CCL23	1.70	62.86	42.43	1.48
NM_002089.1	CXCL2	1.21	56.14	38.96	1.44
NM_203440.2	C10orf4	24.81	28.07	20.07	1.40
NM_005103.3	FEZ1	0.71	19.89	14.37	1.38
NM_145057.2	CDC42EP5	3.43	57.36	43.99	1.30
NM_005239.4	ETS2	0.93	7.20	5.53	1.30
NM_152277.1	DC-UbP	0.54	2.66	2.06	1.29
NM_004591.1	CCL20	0.84	104.40	80.73	1.29
NM_004004.3	GJB2	0.60	27.11	21.01	1.29
NM_007115.2	TNFAIP6	0.62	14.36	11.29	1.27
NM_004172.3	SLC1A3	0.43	18.61	14.70	1.27
NM_002648.2	PIM1	1.00	3.03	2.41	1.26
NM_182648.1	BAZ1A	0.96	2.01	1.61	1.25
NM_006875.2	PIM2	0.89	5.88	4.99	1.18
NM_002638.2	PI3	1.67	372.48	316.93	1.18
NM_014143.2	CD274	0.35	24.97	21.98	1.14
NM_006636.2	MTHFD2	0.84	4.38	3.89	1.12
NM_005080.2	XBP1	0.94	2.27	2.03	1.12
NM_003234.1	TFRC	0.96	3.24	2.92	1.11
NM_004120.3	GBP2	0.98	2.29	2.07	1.11
NM_004776.2	B4GALT5	0.83	4.34	3.93	1.11
NM_002827.2	PTPN1	0.98	1.97	1.78	1.10
NM_022307.1	ICA1	1.00	16.40	14.96	1.10
NM_032793.2	MFSD2	1.77	135.58	124.33	1.09
NM_006399.2	BATF	1.27	16.73	15.44	1.08
NM_004295.2	TRAF4	0.67	38.67	35.73	1.08
NM_213662.1	STAT3	0.89	1.98	1.83	1.08
NM_003955.3	SOCS3	1.46	29.26	27.10	1.08
NM_000610.3	CD44	0.92	3.01	2.80	1.08
NM_006406.1	PRDX4	0.93	2.54	2.37	1.07
NM_016327.2	UPB1	0.83	8.78	8.22	1.07
NM_001710.4	CFB	1.00	308.69	289.22	1.07
NM_000584.2	IL8	0.83	9.76	9.20	1.06
NM_024930.1	ELOVL7	0.73	50.63	50.60	1.00
NM_016229.2	CYB5R2	0.92	234.10	235.57	0.99
NM_000636.2	SOD2	0.45	29.75	30.25	0.98
NM_005558.3	LAD1	0.29	17.20	17.56	0.98
NM_004595.2	SMS	1.02	2.58	2.65	0.97
NM_018993.2	RIN2	0.92	11.13	11.57	0.96
NM_002748.2	MAPK6	0.98	2.38	2.49	0.95

NM_014331.2	SLC7A11	0.09	6.56	6.99	0.94
NM_016354.3	SLCO4A1	1.08	4.78	5.12	0.93
NM_018465.1	C9orf46	1.16	2.60	2.78	0.93
NM_145804.1	ABTB2	1.36	86.94	93.44	0.93
NM_016542.2	MASK	0.98	1.96	2.13	0.92
NM_005746.1	PBEF1	1.04	3.96	4.34	0.91
NM_017819.1	RG9MTD1	1.10	1.62	1.78	0.91
NM_133436.1	ASNS	1.15	1.98	2.18	0.91
NM_003358.1	UGCG	1.10	2.06	2.28	0.91
NM_004998.2	MYO1E	1.00	26.26	29.02	0.91
NM_153186.3	ANKRD15	0.85	12.77	14.22	0.90
NM_005755.2	EBI3	0.54	32.02	35.93	0.89
NM_022767.2	ISG20L1	1.02	3.48	3.91	0.89
NM_000143.2	FH	0.97	2.05	2.31	0.88
NM_000617.1	SLC11A2	0.97	2.72	3.08	0.88
NM_002575.1	SERPINB2	1.05	42.99	49.08	0.88
NM_017746.1	TEX10	1.03	1.62	1.86	0.87
NM_002020.1	FLT4	0.91	2.47	2.84	0.87
NM_014976.1	PDCD11	0.97	1.57	1.81	0.87
NM_016234.3	ACSL5	1.36	23.12	26.70	0.87
NM_014570.3	ARFGAP3	1.02	2.94	3.40	0.86
NM_020117.8	LARS	1.03	1.69	2.00	0.85
NM_024490.2	ATP10A	0.12	2.61	3.08	0.85
NM_002423.3	MMP7	0.32	27.88	33.15	0.84
NM_033050.2	SUCNR1	1.00	40.39	48.47	0.83
NM_032737.2	LMNB2	1.00	1.52	1.86	0.82
NM_004566.2	PFKFB3	1.11	3.64	4.52	0.80
NM_002982.3	CCL2	0.99	40.03	49.83	0.80
NM_015934.3	NOP5/NOP58	0.99	1.78	2.22	0.80
NM_014878.3	KIAA0020	1.00	2.34	2.97	0.79
NM_002161.2	IARS	0.88	1.99	2.53	0.79
NM_032242.2	PLXNA1	0.89	2.01	2.62	0.77
NM_014777.1	KIAA0133	1.32	23.88	31.15	0.77
NM_005371.3	METTL1	1.59	25.28	33.17	0.76
NM_006372.3	SYNCRIP	0.99	1.57	2.11	0.74
NM_006273.2	CCL7	0.82	12.98	17.53	0.74
NM_004728.2	DDX21	0.92	3.34	4.53	0.74
NM_012258.2	HEY1	7.59	93.44	128.00	0.73
NM_001032281.1	TFPI	0.37	14.68	20.27	0.72
NM_004741.1	NOLC1	0.35	6.61	9.18	0.72

Values shown correspond to a ratio determined between normalized gene intensity values obtained after a four-hour PBMC stimulation with either beads, live or lysed Bb (MOI 10:1), in proportion to gene intensity values from unstimulated cells. Live vs lysate ratio is also shown

Supplemental Table 1B: Genes similarly down-regulated by live and lysed *B. burgdorferi* (Bb)

Transcript	Symbol	Beads/Untreated	Live/Untreated	Lysate/Untreated	Live/Lysate
NM_024843.2	CYBRD1	1.22	0.03	0.02	1.47
NM_002612.2	PDK4	1.15	0.04	0.03	1.38
NM_012228.2	MSRB2	1.09	0.55	0.43	1.29
NM_018837.2	SULF2	0.94	0.25	0.21	1.22
NM_016081.2	PALLD	1.22	0.19	0.16	1.22
NM_012387.1	PADI4	1.09	0.57	0.47	1.21
NM_004567.2	PFKFB4	1.20	0.09	0.08	1.19
NM_144687.1	NALP12	1.08	0.04	0.03	1.19
NM_001031835.1	PHKB	0.95	0.63	0.54	1.18
NM_207336.1	ZNF467	1.09	0.03	0.03	1.16
NM_080546.2	SLC44A1	1.05	0.48	0.41	1.15
NM_199418.1	PRCP	1.00	0.46	0.40	1.15
NM_015076.3	CDC2L6	1.02	0.45	0.40	1.15
NM_004695.2	SLC16A5	1.00	0.13	0.11	1.10
NM_006416.2	SLC35A1	1.07	0.67	0.63	1.07
NM_002162.2	ICAM3	1.01	0.65	0.62	1.05
NM_024617.2	ZCCHC6	1.00	0.62	0.60	1.03
NM_002085.2	GPX4	1.07	0.68	0.66	1.03
NM_207514.1	FLJ20186	1.05	0.57	0.55	1.02
NM_002576.3	PAK1	0.94	0.44	0.44	1.02
NM_001031806.1	ALDH3A2	1.05	0.56	0.55	1.01
NM_003331.3	TYK2	1.01	0.56	0.55	1.01
NM_022355.1	DPEP2	0.91	0.27	0.27	1.01
NM_170699.1	GPBAR1	1.29	0.13	0.13	1.00
NM_130768.1	ASZ1	0.09	0.09	0.09	1.00
NM_001466.2	FZD2	0.57	0.02	0.02	1.00
NM_001557.2	IL8RB	1.02	0.02	0.02	1.00
NM_000618.2	IGF1	9.13	0.68	0.68	1.00
NM_015996.1	SIDT2	1.06	0.55	0.55	1.00
NM_014033.2	METTL7A	0.85	0.03	0.03	1.00
NM_152889.1	CHST13	1.70	0.02	0.02	1.00
NM_007278.1	GABARAP	0.99	0.42	0.42	0.99
NM_018353.3	C14orf106	1.00	0.48	0.48	0.99
NM_001020820.1	MYADM	0.86	0.40	0.41	0.97
NM_012199.2	EIF2C1	0.93	0.32	0.33	0.97
NM_001005415.1	39143	1.01	0.42	0.44	0.96
NM_000416.1	IFNGR1	0.98	0.41	0.43	0.96

NM_002746.1	MAPK3	1.10	0.48	0.50	0.96
NM_170709.1	SGK3	1.13	0.48	0.50	0.96
NM_007032.3	HRIHFB2122	1.00	0.29	0.31	0.95
NM_016462.2	TMEM14C	1.03	0.57	0.61	0.95
XM_942125.1	ULK1	1.13	0.41	0.43	0.95
NM_005085.2	NUP214	1.00	0.37	0.39	0.94
NM_000147.2	FUCA1	1.02	0.17	0.18	0.94
NM_016332.2	SEPX1	0.93	0.30	0.32	0.94
NM_032356.3	LSMD1	1.04	0.49	0.53	0.93
NM_000698.2	ALOX5	1.03	0.24	0.26	0.93
NM_003505.1	FZD1	1.39	0.05	0.06	0.92
NM_006834.2	RAB32	1.13	0.56	0.61	0.92
NM_001032296.1	STK24	0.98	0.45	0.49	0.92
NM_139274.1	ACSS2	1.06	0.33	0.36	0.92
NM_004069.3	AP2S1	1.00	0.55	0.61	0.92
NM_001772.2	CD33	1.02	0.29	0.32	0.91
NM_000521.2	HEXB	0.99	0.34	0.38	0.91
NM_014210.2	EVI2A	1.06	0.42	0.47	0.90
NM_003255.4	TIMP2	1.01	0.07	0.07	0.90
NM_134426.1	SLC26A6	1.07	0.22	0.24	0.89
NM_030918.5	SNX27	1.05	0.47	0.53	0.89
NM_018434.4	RNF130	1.00	0.35	0.39	0.89
NM_001005290.2	PSRC1	1.20	0.06	0.07	0.88
NM_015995.2	KLF13	0.99	0.56	0.64	0.88
NM_014604.2	TAX1BP3	0.99	0.43	0.50	0.88
NM_014521.1	SH3BP4	1.08	0.18	0.21	0.86
NM_001012479.1	GRN	1.14	0.27	0.32	0.85
NM_002863.3	PYGL	1.01	0.47	0.56	0.85
NM_021649.3	TICAM2	1.01	0.41	0.49	0.85
NM_024527.3	ABHD8	0.97	0.38	0.45	0.85
NM_001375.2	DNASE2	1.05	0.28	0.34	0.85
NM_201428.1	RTN3	2.37	0.08	0.09	0.84
NM_006432.3	NPC2	1.06	0.46	0.54	0.84
NM_005730.2	CTDSP2	1.12	0.55	0.66	0.84
NM_006120.2	HLA-DMA	0.99	0.39	0.47	0.83
NM_000442.2	PECAM1	1.02	0.12	0.14	0.83
NM_023003.1	TM6SF1	1.00	0.08	0.10	0.83
NM_006195.4	PBX3	1.00	0.52	0.64	0.82
NM_000895.1	LTA4H	1.01	0.24	0.29	0.82
NM_021737.1	CLCN6	1.01	0.49	0.60	0.82
NM_006495.2	EVI2B	1.03	0.33	0.40	0.82
NM_001487.1	BLOC1S1	0.93	0.55	0.69	0.81
NM_002118.3	HLA-DMB	1.08	0.34	0.42	0.81

NM_001064.1	TKT	1.02	0.29	0.36	0.81
NM_014059.1	RGC32	0.98	0.44	0.54	0.81
NM_144641.1	PPM1M	0.93	0.43	0.54	0.80
NM_000358.1	TGFBI	0.90	0.08	0.10	0.80
NM_145008.1	YPEL4	1.08	0.25	0.31	0.80
NM_003405.3	YWHAH	0.95	0.25	0.32	0.78
NM_000647.3	CCR2	1.04	0.02	0.03	0.78
NM_170773.1	RASSF2	0.98	0.41	0.53	0.77
NM_005819.4	STX6	1.00	0.23	0.30	0.77
NM_007074.2	CORO1A	0.96	0.43	0.57	0.76
NM_030912.1	TRIM8	0.92	0.33	0.43	0.75
NM_001909.3	CTSD	1.07	0.25	0.33	0.75
NM_005337.2	HEM1	0.95	0.41	0.55	0.75
NM_002087.2	GRN	1.05	0.25	0.34	0.73
NM_144673.2	CMTM2	1.00	0.25	0.34	0.73
NM_053054.2	CATSPER1	1.05	0.20	0.27	0.72
NM_001323.2	CST6	0.90	0.00	0.00	0.72
NM_006472.1	TXNIP	0.95	0.38	0.53	0.72
NM_145183.1	PYCARD	1.02	0.20	0.28	0.71
NM_005597.2	NFIC	1.03	0.48	0.68	0.71

Values shown correspond to a ratio determined between normalized gene intensity values obtained after a four-hour PBMC stimulation with either beads, live or lysed Bb (MOI 10:1), in proportion to gene intensity values from unstimulated cells. Live vs lysate ratio is also shown
