# Supplementary material for: Activation of Human Monocytes by Live Borrelia burgdorferi Generates TLR2-Dependent and -Independent Responses Which Include Induction of IFN-β
Source: PLoS Pathog. 2009 May 22;5(5):e1000444. doi: 10.1371/journal.ppat.1000444 (PMC2679197; doi:10.1371/journal.ppat.1000444)
Supplement: Table S2 — Genes classified as being down-regulated in peripheral blood mononuclear cells (PBMCs) stimulated with live Borrelia burgdorferi (Bb) MOI (10∶1), in comparison to cells stimulated with similar concentrations of borrelial lysates. (0.10 MB PDF) [file ppat.1000444.s002.pdf]

Supplemental Table 2: Genes classified as being down-regulated in peripheral blood mononuclear cells (PBMCs) stimulated with live *Borrelia burgdorferi* (Bb) MOI (10:1), in comparison to cells stimulated with similar concentrations of borrelial lysates.

Gene Number	Annotation	Beads/UN	Live/UN	Lysate/UN	Live/Lysate	Description/Function
						**Signaling/Transcription Regulation**
NM_002488.2	NDUFA2	1.0	0.7	0.7	0.9	Accessory subunit of the mitochondrial membrane resp. chain
NM_005437.1	NCOA4	1.0	0.6	0.7	0.9	Transmembrane receptor protein tyrosine kinase signaling
NM_001560.2	IL13RA1	1.0	0.1	0.2	0.7	Interleukin 13 receptor, α 1
NM_033297.1	NALP12	1.1	0.1	0.1	0.6	Regulation of interleukin-18 biosynthesis
NM_006068.2	TLR6	1.1	0.0	0.1	0.6	Toll Like Receptor 6
NM_006866.1	LILRA2	1.3	0.2	0.4	0.5	LILRA2 activation inhibits dendrite cell differentiation
						**Cell Activation and/or Cell Cycle**
NM_005211.2	CSF1R	1.00	0.09	0.13	0.7	Transmembrane receptor protein tyrosine kinase signaling
NM_012083.2	FRAT2	1.16	0.44	0.68	0.6	Cell proliferation
NM_004635.3	MAPKAPK3	1.04	0.31	0.48	0.6	Mitogen-activated protein kinase-activated protein kinase 3
NM_003656.3	CAMK1	1.12	0.04	0.06	0.6	Calcium/calmodulin-dependent protein kinase I
NM_001006665.1	RPS6KA1	0.92	0.15	0.27	0.5	ERK activated ribosomal protein S6 kinase
NM_181795.1	PKIB	1.96	0.04	0.35	0.1	Protein Kinase Inhibitor
NM_002775.3	HTRA1	0.95	0.00	0.08	0.05	Regulation of cell growth
						**Cell Damage repair and apoptosis**
NM_001001548.1	CD36	0.88	0.11	0.16	0.7	Scavenger receptor on monocytes
NM_002332.1	LRP1	1.06	0.03	0.05	0.5	Alpha 2 microglobulin receptor (CD91)
NM_015916.3	FAM26B	1.02	0.06	0.27	0.2	Associated with cell viability
						**Ion and Metal Transport**
NM_018404.1	CENTA2	0.90	0.11	0.16	0.6	Metal ion binding
NM_201397.1	GPX1	0.96	0.28	0.44	0.6	Glutathione peroxidase 1 anti oxidant enzyme
NM_002961.2	S100A4	1.01	0.36	0.57	0.6	Calcium ion Binding Protein
NM_002003.2	FCN1	0.98	0.39	0.64	0.6	Calcium ion binding
NM_001623.3	AIF1	0.85	0.34	0.61	0.6	Calcium-binding, inflammation-responsive scaffold protein
NM_000104.2	CYP1B1	0.92	0.23	0.43	0.5	Metal ion binding
NM_138444.2	KCTD12	1.07	0.14	0.28	0.5	Potassium channel tetramerisation domain
NM_002084.2	GPX3	0.94	0.02	0.05	0.4	Selenium binding
NM_030622.6	CYP2S1	1.03	0.02	0.06	0.3	Cytochrome P450 2S1
NM_015481.1	ZNF385	0.91	0.00	0.02	0.2	Metal ion binding
NM_014404.1	CACNG5	0.79	0.03	0.56	0.1	Calcium channel, voltage-dependent, gamma subunit 5
						**Cell architecture**
NM_021647.5	MFAP3L	0.1	0.2	3.1	0.1	Microfibrillar-associated protein 3-like
NM_000423.2	KRT2A	3.0	0.1	2.2	0.05	Structural development of cytoskeleton
NM_031964.1	KRTAP17-1	10.0	0.2	5.7	0.04	Intermediate filament
						**Various Cellular functions**
NM_004893.2	HA2FY	1.1	0.7	0.7	1.0	Nucleosome assembly
NM_001862.2	COX5B	1.0	0.7	0.8	0.9	Nuclear-coded polypeptide chains of cytochrome c oxidase
NM_001152.1	SLC25A5	1.0	0.7	0.9	0.8	Mitochondrial transport
NM_003168.1	SUPT4H1	0.9	0.5	0.8	0.7	Zinc ion binding
NM_024571.2	C16orf33	1.0	0.5	0.8	0.7	Protein modification
NM_031286.2	SH3BGRL3	1.0	0.5	0.8	0.6	Cytoplasmic protein

Gene Number	Annotation	Beads/UN	Live/UN	Lysate/UN	Live/Lysate	Description/Function
						**Phagocytosis/endosomal**
NM_024734.2	CLMN	1.01	0.18	0.27	0.7	Actin binding
NM_005817.3	M6PRBP1	1.04	0.37	0.59	0.6	Vesicle-mediated transport
NM_001671.2	ASGR1	1.04	0.00	0.01	0.3	Mediates endocytosis
NM_138717.1	PPT2	0.04	0.04	0.13	0.3	Lysosomal metabolism
						**Various Cell Metabolism**
NM_030769.1	NPL	1.11	0.31	0.45	0.7	Lygase activity
NM_021626.1	SCPEP1	1.05	0.36	0.53	0.7	Proteolysis and peptidolysis
NM_052960.1	RBP7	0.97	0.19	0.30	0.6	Retinol binding
NM_181873.1	MTMR11	1.04	0.22	0.36	0.6	Phospholipid dephosphorylation
NM_022349.2	MS4A6A	1.16	0.04	0.06	0.6	Signal transduction, integral to membrane
NM_020037.1	ABCC3	0.93	0.12	0.21	0.6	ATPase activity
NM_000714.4	BZRP	1.04	0.27	0.49	0.6	Mitochondrial outer membrane
NM_020400.4	GPR92	0.94	0.29	0.53	0.5	Purinergic nucleotide receptor activity, G-protein coupled
NM_000076.1	CDKN1C	1.06	0.02	0.04	0.5	Regulation of cyclin dependent protein kinase activity
NM_006498.2	LGALS2	1.02	0.14	0.27	0.5	Galectin 2. Sugar binding
NM_145867.1	LTC4S	0.31	0.02	0.05	0.4	Leukotriene biosynthesis
NM_016546.1	C1RL	1.39	0.03	0.07	0.4	Chymotrypsin activity
NM_014449.1	GPR162	1.09	0.02	0.08	0.2	G-protein coupled receptor protein signaling pathway
NM_001030010.1	ALDH3B1	1.08	0.02	0.10	0.2	Aldehyde dehydrogenase [NAD(P)+] activity
NM_017888.2	FLJ20581	0.08	0.08	0.68	0.1	Ligase activity
NM_013402.3	FADS1	0.86	0.04	0.43	0.1	Fatty acid metabolism
NM_174892.1	CD300LB	0.63	0.02	0.23	0.1	Receptor activity
						**DNA RNA metabolism related**
NM_002937.3	RNASE4	0.99	0.01	0.01	0.4	mRNA cleavage
NM_001012329.1	CTNNBIP1	0.74	0.03	0.08	0.3	Regulation of transcription, DNA-dependent
NM_005615.2	RNASE6	1.07	0.02	0.07	0.2	RNA catabolism
NM_000376.2	VDR	1.21	0.02	0.12	0.2	Regulation of transcription, DNA-dependent
NM_021784.3	FOXA2	0.62	0.02	0.71	0.03	Regulation of transcription, DNA-dependent

Values shown correspond to a ratio determined between normalized gene intensity values obtained after a four hour PBMC stimulation with either beads, live or lysed Bb (MOI 10:1), in proportion to gene intensity values from unstimulated cells. Yellow shades highlight genes that were exclusively down-regulated by live Bb.
