# Supplementary material for: Activation of Human Monocytes by Live Borrelia burgdorferi Generates TLR2-Dependent and -Independent Responses Which Include Induction of IFN-β
Source: PLoS Pathog. 2009 May 22;5(5):e1000444. doi: 10.1371/journal.ppat.1000444 (PMC2679197; doi:10.1371/journal.ppat.1000444)
Supplement: Table S3 — Genes more intensely or exclusively up-regulated by lysed B. burgdorferi. (0.06 MB PDF) [file ppat.1000444.s003.pdf]

Supplemental Table 3: Genes more intensely or exclusively up-regulated by lysed *B. burgdorferi*

Gene Number	Annotation	Beads/Untreated	Live/Untreated	Lysate/Untreated	Live/Lysate
NM_017920.2	URG4	0.93	1.20	1.54	0.78
NM_001003796.1	NHP2L1	1.02	1.27	1.73	0.74
NM_022366.1	TFB2M	0.90	1.48	2.11	0.70
NM_006191.1	PA2G4	0.86	1.06	1.55	0.69
NM_001363.2	DKC1	0.97	1.43	2.10	0.68
NM_006303.2	JTV1	0.99	1.72	2.56	0.67
NM_002791.1	PSMA6	0.98	2.44	3.68	0.66
NM_007283.5	MGLL	1.26	4.79	7.25	0.66
NM_001432.1	EREG	0.93	16.20	24.62	0.66
NM_001511.1	CXCL1	0.94	19.32	29.43	0.66
NM_004053.3	BYSL	0.70	2.80	4.28	0.66
NM_000491.2	C1QB	0.77	22.83	36.50	0.63
NM_021205.4	RHOU	0.89	3.62	6.28	0.58
NM_012474.3	UCK2	1.00	1.96	3.44	0.57
NM_014707.1	HDAC9	0.86	22.07	38.83	0.57
NM_000245.2	MET	3.70	22.75	40.11	0.57
NM_198175.1	NME1	0.90	3.17	5.77	0.55
NM_033316.2	MFI2	0.60	18.67	37.43	0.50
NM_001005353.1	AK3L1	1.68	16.38	34.04	0.48
NM_002994.3	CXCL5	1.00	39.15	83.87	0.47
NM_005806.2	OLIG2	1.34	60.21	156.06	0.39
NM_002425.1	MMP10	1.00	88.52	239.19	0.37
NM_001032360.1	MMP19	7.53	26.05	74.70	0.35
NM_153836.1	CREG2	10.02	1.06	3.10	0.34
NM_003713.3	PPAP2B	1.00	10.64	33.57	0.32
NM_002815.2	PSMD11	1.00	10.94	38.69	0.28
NM_002421.2	MMP1	0.60	9.15	34.70	0.26
NM_000930.2	PLAT	1.00	19.05	133.90	0.14
NM_181505.1	PPP1R1B	1.00	1.00	9.38	0.11
NM_021647.5	MAFP3L	0.06	0.17	3.10	0.06
NM_000423.2	KRT2A	2.99	0.10	2.20	0.05
NM_031964.1	KRTAP17-1	10.00	0.23	5.70	0.04
