# Supplementary material for: Activation of Human Monocytes by Live Borrelia burgdorferi Generates TLR2-Dependent and -Independent Responses Which Include Induction of IFN-β
Source: PLoS Pathog. 2009 May 22;5(5):e1000444. doi: 10.1371/journal.ppat.1000444 (PMC2679197; doi:10.1371/journal.ppat.1000444)
Supplement: Table S4 — Differentially regulated type I interferon associated genes. (0.07 MB PDF) [file ppat.1000444.s004.pdf]

Supplemental Table 4. Differentially Regulated Type I Interferon Associated Genes

Up Regulation (live or lysed vs. unstimulated)			
Symbol	Live Bb	Lysed Bb	Description
CNTFR	5.7	1.5	Ciliary neurotrophic factor receptor
CXCL10	9.7	-1.1	Chemokine (C-X-C motif) ligand 10
EBI3	28.3	24.0 *	Epstein-Barr virus induced gene 3
F3 **	169.4	21.8 *	Coagulation factor III (thromboplastin, tissue factor)
ISG15 **	7.1	-1.9	ISG15 ubiquitin-like modifier
IFI27	12.7	4.8 *	Interferon, alpha-inducible protein 27
IFI35 **	4.9	1.3	Interferon-induced protein 35
IFIT1 **	7.8	-1.6	Interferon-induced protein with tetratricopeptide repeats 1
IFIT2 **	4.3	-2.5	Interferon-induced protein with tetratricopeptide repeats 2
IFIT3	5.9	-2.1	Interferon-induced protein with tetratricopeptide repeats 3
IFNA6	8.5	4.4 *	Interferon, alpha 6
IFNB1 **	21.2	2.4	Interferon, beta 1
IFNK	6.8	2.8	Interferon, kappa
IFRD2	5.6	6.2 *	Interferon-related developmental regulator 2
IL12B **	372.6	14.5 *	Interleukin 12B
IL28RA	5.6	2.0	Interleukin 28 receptor, alpha
IL6 **	3376.5	665.1 *	Interleukin 6
IL7R	11.9	11.5 *	Interleukin 7 receptor
MX1 **	5.1	-1.2	Myxovirus (influenza virus) resistance 1

Down Regulation (live or lysed vs. unstimulated)			
Symbol	Live Bb	Lysed Bb	Description
IL13RA1 **	-7.0	-5.1*	Interleukin 13 receptor, alpha 1
IRF2BP2	-6.0	-3.5	Interferon regulatory factor 2 binding protein 2

* Indicates Response was also significant for lysed Bb stimulated monocytes

** Indicates Response was also differentially regulated in the PBMC array
